# Leveraging universal and transfer learning models for influenza prediction in Thailand

**DOI:** 10.1038/s41598-026-37855-7

**Published:** 2026-01-30

**Authors:** Pitiwat Lueangwitchajaroen, Suparinthon Anupong, Chanidapa Winalai, Sudarat Chadsuthi

**Affiliations:** 1 Department of Physics and Astronomy, University of Nottingham, Nottingham, UK; 2 Faculty of Science, Naresuan University, Phitsanulok, 65000 Thailand; 3 University Grenoble Alpes, CNRS, UMR 5525, VetAgro Sup, Grenoble INP, TIMC, Grenoble, 38000 France; 4 Department of Physics, Faculty of Science, Naresuan University, Phitsanulok, 65000 Thailand

**Keywords:** Transfer learning, Neural network, Deep learning, Influenza, Infectious diseases, Computational science

## Abstract

Influenza is a major respiratory disease that causes significant morbidity and mortality worldwide. Accurate predictions of influenza incidence enable public health organizations to monitor and prepare for outbreaks, ultimately reducing mortality and optimizing resource allocation. However, many countries, including Thailand, face challenges in generating accurate forecasts due to limited feature data in certain regions. To address this, we developed universal deep learning (DL)-based models to predict influenza incidence across multiple provinces in Thailand from 2010 to 2019. We evaluated various model configurations and implemented a feature selection process to enhance model generalizability and performance by ensuring equal contributions from multiple time series features. Our findings indicate that single hidden layer models with 128 nodes performed the best in the universal framework. To extend predictions to provinces without meteorological and PM10 data, we applied transfer learning (TL) using pre-trained models. The TL-based model, fine-tuned for each province, significantly outperformed baseline models trained solely on previous incidence, achieving the highest accuracy. Our results demonstrate the potential of universal DL and TL frameworks in forecasting influenza trends, even in limited data regions, and highlight the importance of incorporating domain-specific knowledge for robust epidemic management strategies.

**Supplementary Information:**

The online version contains supplementary material available at 10.1038/s41598-026-37855-7.

## Introduction

Influenza is a respiratory disease that causes significant morbidity and mortality each year and remains a crucial public health concern^1^. The 2009 H1N1 pandemic led to approximately 201,200 respiratory-related deaths and an additional 83,300 deaths due to cardiovascular complications^2^. Nearly 80% of these fatalities occurred in individuals under 65 years of age^2^. Southeast Asia and Africa were particularly affected, accounting for 51% of these cases^2^. While the number of influenza cases declined during the COVID-19 pandemic due to non-pharmaceutical interventions^3^, recent influenza seasons have shown a resurgence, with the 2022–2023 season in the United States classified as moderate in severity^4^.

Various studies have highlighted the potential role of climate factors in influencing seasonal influenza transmission. In Thailand, the seasonal behavior of influenza may be influenced by a range of climate factors due to the country’s diverse environment. For example, using a Seasonal Autoregressive Integrated Moving Average (SARIMA) model, a study in Bangkok found that increased influenza activity was linked to increased relative humidity^5^. In the central region of Thailand, both average temperature and minimum relative humidity correlated with influenza cases, while in the southern region, only average temperature was associated with influenza using the ARIMA model^6^. Additionally, higher particulate matter (PM) concentrations were associated with increased influenza incidence in Chiang Mai^7^.

Traditional statistical models, such as ARIMA and Poisson regression, have been widely used to capture seasonal disease trends and analyze incidence. However, these models often require region-specific training and may not be accurate to capture irregular outbreaks or non-seasonal trends, limiting their scalability across multiple regions^8,9^.

Recent advances in artificial intelligence have led to the increased application of machine learning (ML) models in predicting infectious diseases. Tree-based models, such as Random Forests (RF) and XGBoost (XGB), have been applied to predict infection rates for multiple diseases^10^. A two-dimensional hierarchical approach using RF and XGB models has also shown promising results in predicting influenza outpatient visits^11^.

Deep learning (DL) models, known for their ability to learn from complex and noisy datasets, have outperformed statistical models in forecasting infectious diseases such as Influenza^12^, COVID-19^13,14^, and Ebola^15^. Recurrent neural networks (RNNs) and their variants, such as long short-term memory (LSTM) networks, have demonstrated high performance in modeling sequential data and predicting future outbreaks^16–18^. For example, a multi-time series LSTM has been proposed to predict future COVID-19 cases and deaths in the USA, demonstrating high performance and reducing computational time compared to standard ensemble methods^13^. However, these DL-based models typically require large amounts of training data and may need retraining for each new region, limiting for large-scale applications^19^.

Transfer learning (TL) has emerged as a promising solution for regions with limited data. This approach involves pre-training a model on data-rich regions and fine-tuning it for regions with limited data. This technique has successfully forecasted and predicted COVID-19^19^. TL is also used to forecast new diseases by pre-training on related diseases and fine-tuning with target disease data^20^. However, its application to influenza forecasting, particularly across diverse regions in Thailand^21^, remains unexplored.

In this study, our primary objective is to develop a transfer learning model to predict influenza incidence in provinces with limited or unavailable feature data. We used meteorological data and PM10 concentrations to construct a universal neural network model that predicts influenza incidence across 22 out of 76 provinces in Thailand from 2010 to 2019^13^. We also evaluated the influence of meteorological and PM10 features on model performance to understand their predictive significance. Our proposed framework is designed to reduce computational time during the training process and facilitate rapid responses to influenza outbreaks. Moreover, the TL framework is adaptable for areas lacking feature data by pre-training the model with data-rich areas, thus supporting evidence-based public health decisions.

## Methods

### Data collections and processing

**Fig. 1:**
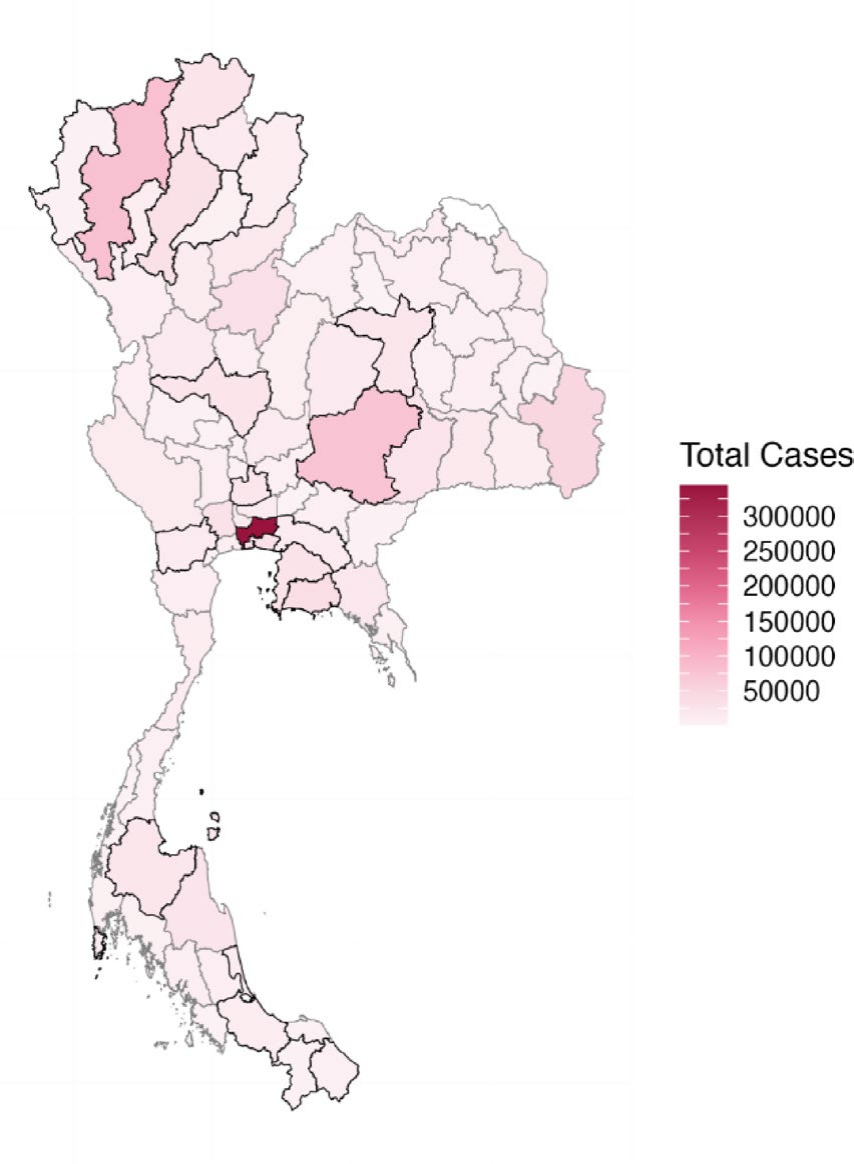
Map of total influenza cases in Thailand between 2010–2019. The highlighted 22 provinces, for which meteorological and PM10 concentrations are available, are indicated by bold black borders.

We obtained monthly influenza case reports from the National Disease Surveillance (Report 506) of the Department of Disease Control, Thailand, from January 2010 to December 2019. These reports included suspected, probable, and confirmed cases, classified according to the communicable disease surveillance case definitions^22^. The provincial total influenza cases, spanning from northern to southern Thailand, are shown in Fig. 1. Daily meteorological data were obtained from the Global Surface Summary of the Day (GSOD) using the R package ‘GSODR’^23^. Moreover, we retrieved PM10 monthly data from the Pollution Control Department, Thailand^24^. Table 1 describes all meteorological data and PM10 for our study.

Of the 76 provinces in Thailand, meteorological and PM10 data were available for only 22 provinces (see the highlighted provinces in Fig. 1), whereas they were unavailable for the remaining 54 provinces. Figures S2-S8 illustrate the monthly time series of influenza incidence, meteorological factors, and PM10 concentrations, respectively, for these 22 provinces. We converted the monthly reported influenza cases to incidence as the number of reported cases divided by the monthly provincial population, multiplied by 10,000, as shown in the box-and-whisker plot in Figure S1. The monthly population for each province was retrieved from the Office of Registration Administration, Department of Provincial Administration. To align a monthly timescale across all variables, we averaged daily meteorological data at each station. Then we averaged station-level values within each province to obtain province-level meteorological features. For monthly PM10 data, we similarly averaged across stations within each province to get a provincial PM10 value for each month. Due to the missing values, we preprocessed PM10 by cleaning the series and imputing missing values using smoothed interpolation.

In this study, we considered lag times for each feature, including meteorological data, PM10 concentrations, and incidence, to capture delayed effects at the monthly time scale. Previous studies in Thailand have shown that meteorological factors can influence influenza activity with delays of approximately 1–4 months^5,6^. Therefore, we included lagged meteorological variables up to 3 months to reduce over-parameterization while capturing the most plausible delayed effects. We applied the same 1–3-month lag window to PM10 to allow for delayed associations and to maintain a consistent feature set across provinces. Finally, we included lagged incidence terms (1–3 months) because influenza incidence may correlate with the recent past incidence, which can improve prediction. All predictors were standardized using Z-score normalization (subtracting the mean and dividing by the standard deviation).

Using data from the 22 provinces with complete features, we trained a universal model. Then we developed a transfer learning (TL) model based on the universal model to generate predictions for the remaining 54 provinces. The final 17 months of observations from each province were held out for model evaluation.

**Table 1:** Description of the features.

Parameters	Descriptions	References
Temperature	Average temperature (^o^C)	^23^
Relative Humidity	Average relative humidity; RH (%)	^23^
Precipitation	Average precipitation (mm)	^23^
Wind Speed	Average wind speed (m/s)	^23^
Visibility	Average visibility (km)	^23^
PM10	Average concentration of particulate matter that less than 10 microns (µg/m^3^)	^24^

### Feature selection

**Fig. 2:**
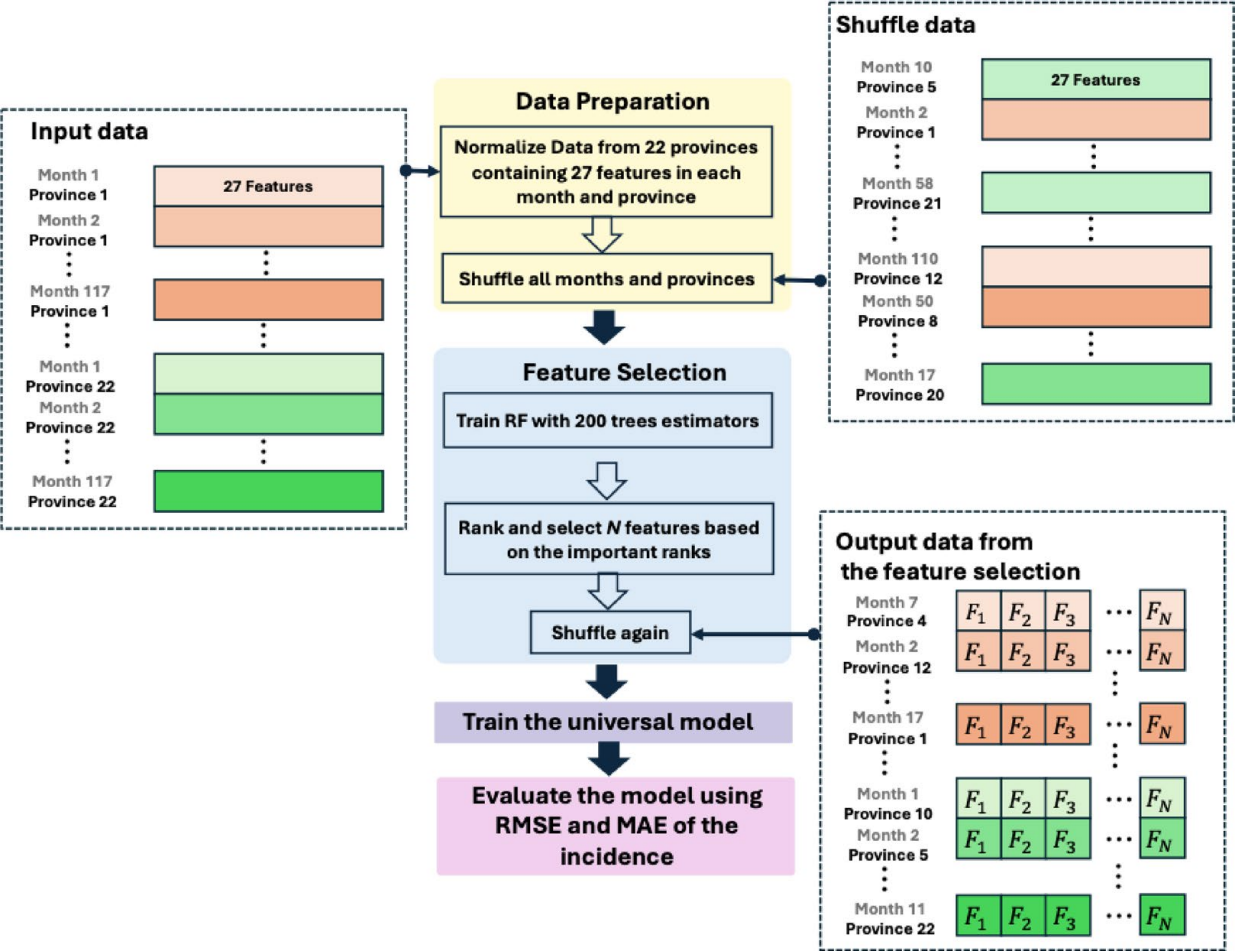
Workflow process for feature selection of the M2 model, starting from data preparation to train the model.

We selected input features to develop an effective universal model using Random Forest (RF)^25^ from the Scikit-learn 1.2.2 library. This approach reduced computational time and improved model performance by identifying features that contribute to the reported incidence data. Figure 2 illustrates the feature selection and model training process for predicting incidence rates.

First, the set of data (Month_i_, Province_k_, Feature_i, k,1_,…, Feature_i, k,27_) was shuffled to create an unbiased dataset and prevent any single feature from dominating due to its scale. This step ensures that all features contribute equally to the model. This method effectively combines temporal (time steps) and spatial (different provinces) data, allowing the model to learn patterns across both dimensions. Such a method is particularly useful because incidence may be influenced by both time- and location-specific factors.

To train the model for the 22 provinces, we constructed Model 1 (M1) as a baseline and used only the previous incidence with three time lags as input features, without environmental variables or feature selection. Model 2 (M2) was constructed using 27 candidate features: lagged meteorological and PM10 variables at 0–3 months and lagged influenza incidence at 1–3 months, as described in Table 1. These features were used in the RF model to identify the most relevant features.

We trained an RF model with 200 tree estimators using the shuffled data^13^. During training, the model used random subsets of the 27 features to build decision trees for the M2 model. After training, the importance of each feature was ranked based on its contribution to the model’s performance. Since the RF model can be sub-optimal and the selected features may vary with each run due to randomness, we conducted 50 experimental runs. For each RF run, we selected $\:N$ features that had an importance score greater than the mean importance score, denoted as $\:{F}_{1},\dots\:,{F}_{N}$. These selected features were used as the input feature set to train a corresponding M2 model. Before training the M2 model, we shuffled the province–month data again to minimize any ordering effects during optimization. This workflow ensures that the most relevant features are included in the universal model, potentially enhancing its accuracy and effectiveness in predicting incidence rates.

### Universal DL-based model

To construct the universal deep learning (DL) model, we used an artificial neural network (ANN), a foundational DL model. An ANN is composed of three main types of layers: an input layer, one or more hidden layers, and an output layer. Each layer contains nodes that apply non-linear activation functions to process information. The connection strengths between nodes in adjacent layers are represented by weights, which determine the significance of the connections. During training, the ANN adjusts the weights to minimize prediction errors^26^.

The structure of the ANN model used in this study is illustrated in Figure S9. The input features are passed to the first dense layer containing $\:n$ nodes. The output from this layer is then passed to subsequent hidden layers. We tested configurations with either one or two hidden layers to identify the optimal model. For a single hidden layer, we tested 8, 16, 32, 64, and 128 nodes. For two hidden layers, we used configurations (8, 8), (16, 16), and (32, 32), where the first value indicates the number of nodes in the first hidden layer, and the second value represents the number of nodes in the second hidden layer.

Each model was regularized using batch normalization after the input layer, followed by ReLU (rectified linear unit) activation functions to capture non-linear relationships in the data. For models with two hidden layers, the output of the first hidden layer was passed to the second hidden layer before being passed to a single node that produced the final regression output. For models with one hidden layer, the output was fed directly to the regression node. The architecture for a single hidden layer can be expressed as follows^27^:

1 $$\:{z}_{1}=\:f\left({W}_{1}^{T}x+{b}_{1}\right)$$

2 $$\:{z}_{2}=\:f\left({W}_{2}^{T}{z}_{1}+{b}_{2}\right)$$

where $\:x$ is the input feature vector, $\:{W}_{1}$ and $\:{W}_{2}$ are weight matrices, $\:{b}_{1}$ and $\:{b}_{2}$ are bias vectors. $\:f$ is an activation function. $\:{z}_{1}$ and $\:{z}_{2}$ represent the output from the first and second layers, respectively.

We trained the ANN model on data from the first 100 months for all 22 provinces, resulting in a total of 2,200 instances. The last 17 months of data from each province were reserved for model evaluation. To investigate the impact of meteorological and PM10 features on model performance, we compared the performance with two universal ANN models. Model 1 (M1) used only the previous incidence as input features (three-time lags of incidence), whereas Model 2 (M2) used the features selected by the RF model.

In this work, we also compare the results of universal ANN models with machine learning models, i.e., linear regression (LR) and XGBoost. These models were selected to represent two distinct modeling assumptions: linearity and non-linearity of predictive ability. LR serves as the baseline for this study. We used Scikit-learn 1.2.2 library for LR with typical ordinary least squares for LR parameter fitting. We selected XGBoost to serve as the non-linear baseline. We used xgboost 1.7.6 library trained with MSE loss, estimators = 1000 estimators, and max_depth = 6.

### Transfer learning model

In our study, meteorological and PM10 features were available for only 22 out of 76 provinces, while incidence was available for all provinces. Our goal was to develop transfer learning (TL) models based on the best-performing universal model to predict incidence for the remaining 54 provinces. We proposed two strategies for TL models (T1 and T2 models), compared with the baseline model (T0 model).

For T0, we applied the universal model M1 to the 54 provinces using only lagged incidence (1–3 months) as inputs, without updating any model parameters. T1 is a pooled fine-tuning strategy. We fine-tuned a single TL model using the combined incidence data from all 54 provinces, and then generated predictions for each province. T2 is a province-specific fine-tuning strategy. We fine-tuned separate models for each province, using only that province’s historical incidence data, resulting in 54 fine-tuned models.

To construct the transfer learning model, we began by introducing the notations. Each province, $\:{P}_{k}$, is defined as:

$$\:{P}_{k}=\left\{\left({x}_{k,t},{y}_{k,t}\right)\right|\:t\in\:\{1,\dots\:,117\}\},\:k=1,\dots\:,76$$

where $\:{y}_{k,t}\in\:\mathbb{R}$ denotes the influenza incidence in province $\:k$ at month $\:t$ (the prediction target), and $\:{x}_{k,t}$ denotes the predictor vector at month $\:t$, may include lagged incidence. For 22 provinces, $\:{x}_{k,t}$ may include meteorological and PM10 features alongside the lag times of incidence. Let $\:\mathcal{D}=\left\{{P}_{k}\right\}$ represent the collection of datasets from the 76 provinces.

We denote the 22 provinces with environmental covariates as group $\:A$ and the remaining 54 provinces as group $\:B$. We divided $\:\mathcal{D}$ into training ($\:{\mathcal{D}}^{\mathrm{t}\mathrm{r}\mathrm{a}\mathrm{i}\mathrm{n}})\:$and testing data set ($\:{\mathcal{D}}^{\mathrm{t}\mathrm{e}\mathrm{s}\mathrm{t}}$). The training dataset $\:{\mathcal{D}}^{\mathrm{t}\mathrm{r}\mathrm{a}\mathrm{i}\mathrm{n}}$ consists of $\:{\mathcal{D}}_{A}^{\mathrm{t}\mathrm{r}\mathrm{a}\mathrm{i}\mathrm{n}}$ (from 22 provinces) and $\:{\mathcal{D}}_{B}^{\mathrm{t}\mathrm{r}\mathrm{a}\mathrm{i}\mathrm{n}}$ (from 54 provinces), while the testing dataset $\:{\mathcal{D}}^{\mathrm{t}\mathrm{e}\mathrm{s}\mathrm{t}}$ consists of $\:{\mathcal{D}}_{A}^{\mathrm{t}\mathrm{e}\mathrm{s}\mathrm{t}}$ and $\:{\mathcal{D}}_{B}^{\mathrm{t}\mathrm{e}\mathrm{s}\mathrm{t}}$, representing the last 17 months for each province. For the T0 model, $\:{\mathcal{D}}_{B}^{\mathrm{t}\mathrm{r}\mathrm{a}\mathrm{i}\mathrm{n}}$ (data from the 54 provinces) is used instead of $\:{\mathcal{D}}_{A}^{\mathrm{t}\mathrm{r}\mathrm{a}\mathrm{i}\mathrm{n}}$ (data from the 22 provinces).

**Fig. 3:**
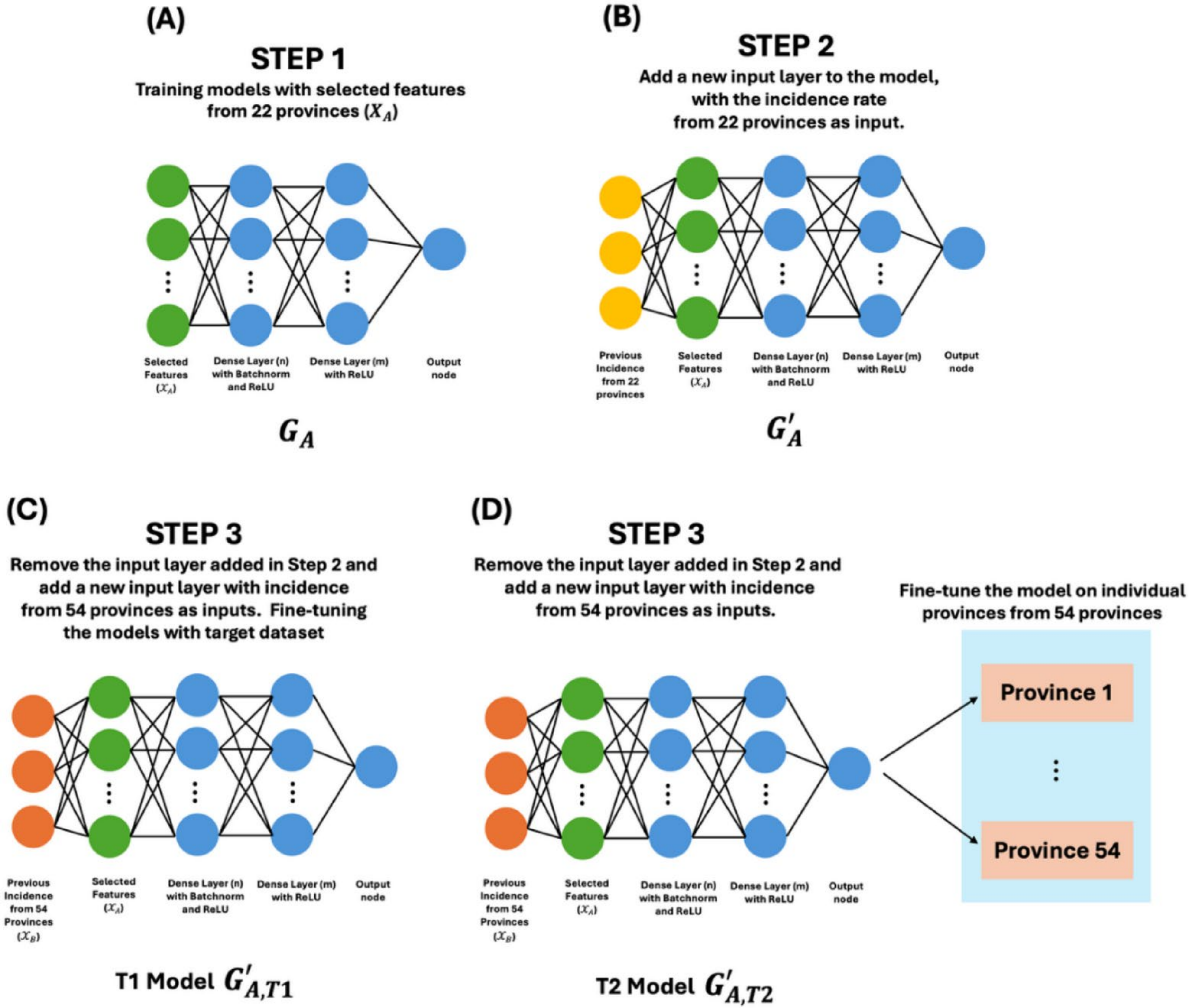
The proposed TL model with three-step process. Step 1 involves training the model denoted as $\:{G}_{A}$ with input features from $\:{\mathcal{D}}_{A}^{\mathrm{t}\mathrm{r}\mathrm{a}\mathrm{i}\mathrm{n}}$ using $\:{\mathcal{X}}_{A}$ (A). In step 2, a new input layer is added to the previous model, denoted as $\:{G}_{A}^{{\prime\:}}$ (B). Step 3 applies a typical transfer learning process from step 2, with two proposed strategies denoted as $\:{G}_{A,T1}^{{\prime\:}}$, and $\:{G}_{A,T2}^{{\prime\:}}$ for the T1 (C) and T2 (D) models, respectively.

Before training the TL models, we used a Random Forest (RF) to select the features from $\:{\mathcal{D}}_{A}^{\mathrm{t}\mathrm{r}\mathrm{a}\mathrm{i}\mathrm{n}}$, as shown in Fig. 2. Here, $\:{\mathcal{X}}_{A}$ is denoted the selected features for $\:{\mathcal{D}}_{A}^{\mathrm{t}\mathrm{r}\mathrm{a}\mathrm{i}\mathrm{n}}$ and $\:{\mathcal{X}}_{B}$ represents the fixed set of features (lagged incidence) for $\:{\mathcal{D}}_{B}^{\mathrm{t}\mathrm{r}\mathrm{a}\mathrm{i}\mathrm{n}}$. We assumed that the distribution of the data in $\:{\mathcal{D}}_{A}$ and $\:{\mathcal{D}}_{B}$ were similar to each other since the data was selected in the same country. The TL model leveraged learned representations from both datasets to improve its performance. We designed the three steps training (Fig. 3) as follows:

- **Step 1:** We trained an ANN regressor $\:{G}_{A}$ on $\:{\mathcal{D}}_{A}^{\mathrm{t}\mathrm{r}\mathrm{a}\mathrm{i}\mathrm{n}}$ using features $\:{\mathcal{X}}_{A}$ (selected features from 22 provinces), as shown in Fig. 3A.
- **Step 2:** A new input layer was added to the trained $\:{G}_{A}$, denoted $\:{G}_{A}^{{\prime\:}}$. We then re-trained $\:{G}_{A}^{{\prime\:}}$ on $\:{\mathcal{D}}_{A}^{\mathrm{t}\mathrm{r}\mathrm{a}\mathrm{i}\mathrm{n}}$ using only incidence of $\:{\mathcal{D}}_{A}^{\mathrm{t}\mathrm{r}\mathrm{a}\mathrm{i}\mathrm{n}}$ so that the model is ready to apply transfer learning to $\:{\mathcal{D}}_{B}^{\mathrm{t}\mathrm{r}\mathrm{a}\mathrm{i}\mathrm{n}}$, as shown in Fig. 3B.
- **Step 3:** We noted that the input node of $\:{G}_{A}^{{\prime\:}}$ was equal to 3. Thus, we can fine-tune the model again on $\:{\mathcal{D}}_{B}^{\mathrm{t}\mathrm{r}\mathrm{a}\mathrm{i}\mathrm{n}}$ using $\:{\mathcal{X}}_{B}$(lagged incidence) from the 54 provinces, as shown in Fig. 3C and D. T1 and T2 model strategies are applied in this step. The T1 model $\:{G}_{A,\:T1}^{{\prime\:}}$ is fine-tuned using incidence data from all 54 provinces (Fig. 3C). The T2 model $\:{G}_{A,\:T2}^{{\prime\:}}$ is fine-tuned individually for each province. The T2 model was trained and tested on data from a specific province and needed re-training for other provinces (Fig. 3D). This independent fine-tuning process was repeated 54 times for all provinces. The performances of the TL models were compared and used to evaluate the accuracy of incidence rate predictions across the 54 provinces.

### Training strategy

We trained the model using the ADAM optimizer^28^ with an initial learning rate of 0.001. The entire dataset was fed into the model at once, without splitting it into batches. At the end of each epoch, 10% of the training data was randomly sampled to create a validation set, which was used for early stopping and model selection. Specifically, if the validation loss did not improve for three consecutive epochs, the learning rate was reduced by a factor of 0.6. If no further improvement occurred after an additional 10 epochs, the training process was automatically stopped. The model with the lowest validation loss was selected as the best-performing model. We evaluated the model’s performance using Root Mean Square Error (RMSE) and Mean Absolute Error (MAE) metrics. The models were compared based on how well they fit the incidence rates.

### Ethics statement

All information on influenza surveillance data was collected from the Division of Epidemiology, Department of Disease Control, the Ministry of Public Health, Thailand. This study was approved by the Institutional Review Board of Naresuan University as Exemption Review (IRB No. P1-0075/2566). The need for informed consent was waived by the Institutional Review Board of Naresuan University as all data from our study are deidentified. All methods were performed in accordance with the relevant guidelines and regulations.

### Software

The code was tested with Python 3.7 and PyTorch 1.12.1 and XGBoost. For the plots, maps, and data analysis, we used R program version 4.3.0^29^ with package tidyverse 2.0.0^30^, ggpubr 0.6.0^31^, spdep 1.2-8^32^, and sf 1.0–13^33^.

## Results

### Universal models

**Fig. 4:**
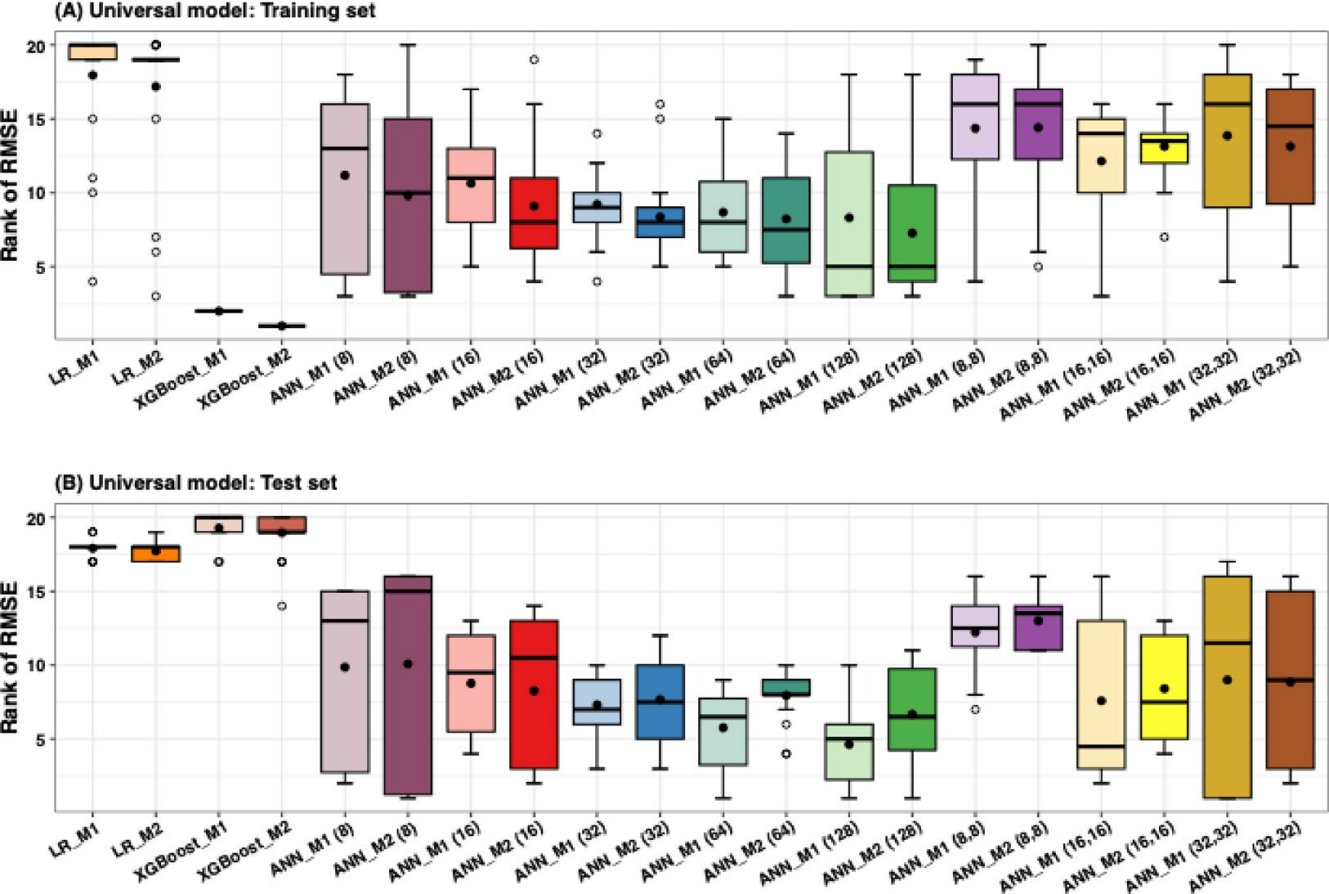
Universal models with RMSE rankings from 50 experimental runs. The rankings are based on the relative ranks for the lowest average RMSEs in the training (**A**) and testing (**B**) sets for 22 provinces. The box plots indicate the 25th and 75th percentiles, while the whiskers represent the maximum and minimum ranks. Black dots and blank circles represent mean values and outliers, respectively. The numbers ($\:n$) and ($\:n,m$) indicate the number of hidden units for single-layer and double-layer of ANN models, respectively.

We constructed the universal LR, XGB, and ANN models using available data from 22 provinces (as described in Fig. 1). This model employed selected features derived from 50 runs of the RF selection process. The feature selection results indicated that PM10 concentration did not play a significant role in predicting influenza incidence across all provinces (Figure S10). Therefore, PM10 concentration was excluded as a feature. We further used the selected features from the 50 experimental runs. The average RMSE and MAE values for both the training and test sets for the 22 provinces are presented in Tables S1-S4. The RMSEs and MAEs were ranked from lowest to highest values, with the first rank (one) representing the best model and the last rank (twentieth) representing the worst. The rankings of RMSEs (and MAEs) for the 22 provinces are illustrated as box-and-whisker plots (showing the interquartile range, minimum, and maximum ranks) in Fig. 4 and Figure S11. The black dots and lines inside the boxes represent the average and median ranks for all models across 20 configurations with different methods and node counts for single ($\:n$) and double ($\:n,m$) hidden layers in the ANN universal model.

For training results (Fig. 4A), the XGB model outperformed, as indicated by lower ranks, while LR had the worst performance. For the ANN models, the single-hidden-layer models consistently outperformed double-hidden-layer models. Overall, adding meteorological features (M2) slightly improved performance. Among the ANN results, the model with the highest number of hidden nodes (128) was the best-performing model, with a median RMSE ranking of 5 for both M1 and M2. The M1 (64) model tied for third place, with median rankings close to 7.5. In contrast, the double hidden layer models showed similar performance between models with (M2) and without (M1) meteorological features. The M1 (16, 16) and M2 (16, 16) models outperformed all other double hidden layer models, both having a median ranking of 11.

For the testing results in Fig. 4B, LR and XGB show the weakest performance. XGBoost underperformed in the test set, which is prone to overfitting. For the ANN models, the results show a similar trend to the training results, with single-hidden-layer models outperforming double-hidden-layer models, except for the (16, 16) model. However, the inclusion of meteorological features (M2) did not consistently enhance performance in the test set for either single or double hidden layer models; in some cases, performance did not improve. Among the ANN models, the M1 (16,16) model achieved the best performance in the test set, with a median ranking of 4.5. The M1 (128) model ranked second, while the M1 (64) and M2 (128) models ranked third, with median rankings of 5 and 6.5, respectively. Overall, we found that the number of hidden units, particularly 64 and 128, influenced performance, with the 128-unit models performing slightly better.

**Fig. 5:**
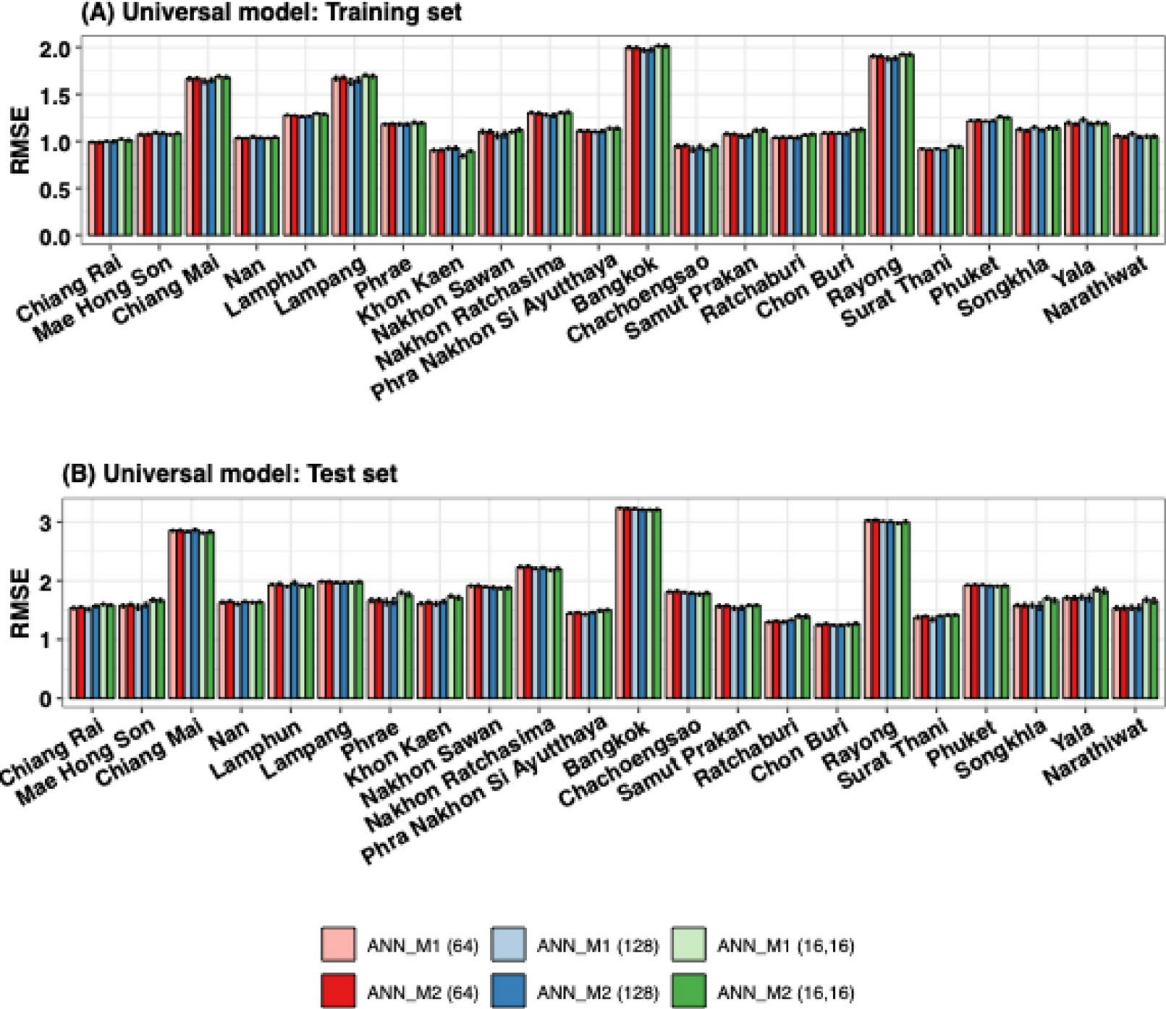
Average RMSE values for the training (**A**) and testing (**B**) sets of the universal models for 22 provinces over 50 runs. The error bars represent the 95% confidence intervals, where lower values indicate better model performance.

Based on all the training and testing results, we selected the universal ANN models with 64 and 128 nodes for the single hidden layer configuration and the model with (16, 16) nodes for the double hidden layer configuration. The average RMSEs (and MAEs) of these models, with and without meteorological features, are shown in Fig. 5 (and Figure S12).

Figure 5A shows low RMSE values for the training sets across most provinces, indicating good model performance, except for Chiang Mai, Lampang, Bangkok, and Rayong, which had slightly higher RMSEs than other provinces. These provinces have higher average incidence rates compared to other provinces. The RMSE values for the M1 model with the single hidden layer were approximately the same as those for the M2 model, with a slight advantage for M2 observed in most provinces. In contrast, the M1 models with the double hidden layer performed slightly better than the M2 models.

For the test set (Fig. 5B), the RMSE values were generally higher than those for the training set, indicating a higher prediction error for unseen data. Models with double hidden layers (M1 (16, 16) and M2 (16, 16)) exhibited higher RMSEs compared to the models with single hidden layers. These results suggest that the double hidden layer models may have underfit the training data, making them less effective at generalization. The single hidden layer models with 128 hidden units (for both M1 and M2) performed the best, as evidenced by the lower RMSE values across most provinces in both the training and testing sets. In the single hidden layer models, no significant improvement was observed in most provinces when meteorological features were included (M2) compared to models trained without them (M1). Overall, the M2 model with 128 hidden units consistently showed better performance, while the M1 model with 128 hidden units highlighted the best test performance across most provinces. We compared the fitted and predicted incidence using the best-performing models (16,16) hidden units for M1 and M2 with the actual data in Figure S13. The results show that the universal model can fit the incidence well across provinces, capturing the overall pattern. However, the fitted curves tend to underestimate peak magnitude in high-incidence provinces, such as Bangkok, Chiang Mai, and Rayong.

### Transfer learning model

We employed transfer learning (TL) models to extend the features from 22 provinces to the other 54 provinces, where meteorological and PM10 features were unavailable. The TL models were trained using two strategies: fine-tuning based on the entire 54 provinces (T1) and province-specific fine-tuning (T2), as described in Sect. 2.4. The universal T0 model was trained solely on the previous incidence rates for the 54 provinces, resembling the M1 model. In this work, we continued analyzing the ANN configurations with both single and double hidden layers. The single-layer models included 64 and 128 hidden units, selected based on their performance in the universal model. The chosen double hidden layer configuration was (16, 16), as it demonstrated the best performance among the double-layer models.

**Fig. 6:**
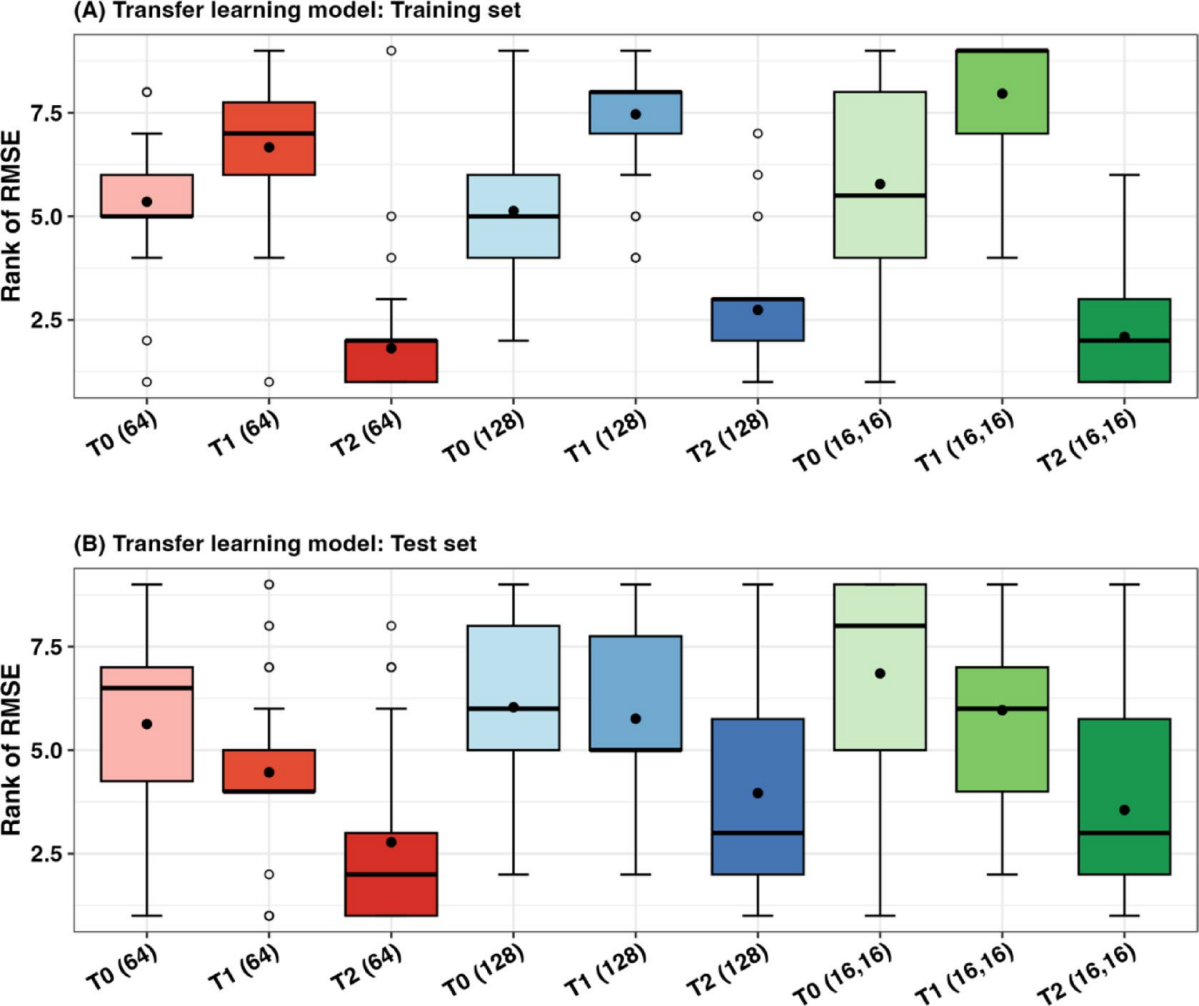
RMSE rankings for the training (**A**) and testing (**B**) sets for all transfer learning models (T0, T1, and T2) from 50 runs. Box-and-whisker plots are shown, with black dots indicating the average RMSE rankings and blank circles indicating outliers.

We evaluated the TL models using RMSEs and MAEs by ranking the models from best (ranked one) to worst (ranked nine), as shown in Fig. 6 and Figure S14, respectively. In the training set (Fig. 6A), the T2 model outperformed the T0 and T1 models. Specifically, the T2 model with 64 hidden units achieved the lowest mean RMSE rank, indicating high performance during training. The T2 models with 128 hidden units and (16, 16) hidden units also performed well. For the test set (Fig. 6B), consistent with the training results, the T2 model with 64 hidden units maintained the lowest mean RMSE rank, demonstrating strong performance on unseen data. Interestingly, we found that the T1 model outperformed T0, suggesting that transfer learning can be effectively applied to predict incidence for provinces with unavailable environmental feature data. For the configurations, the T2 model with 64 hidden units performs better than the T2 (128) and T2 (16, 16), particularly in the test set. In contrast, the T2 (16, 16) model often shows significantly higher RMSE rank than the single hidden layer models with 64 units. This suggests that increasing model complexity does not necessarily enhance its ability to generalize.

**Fig. 7:**
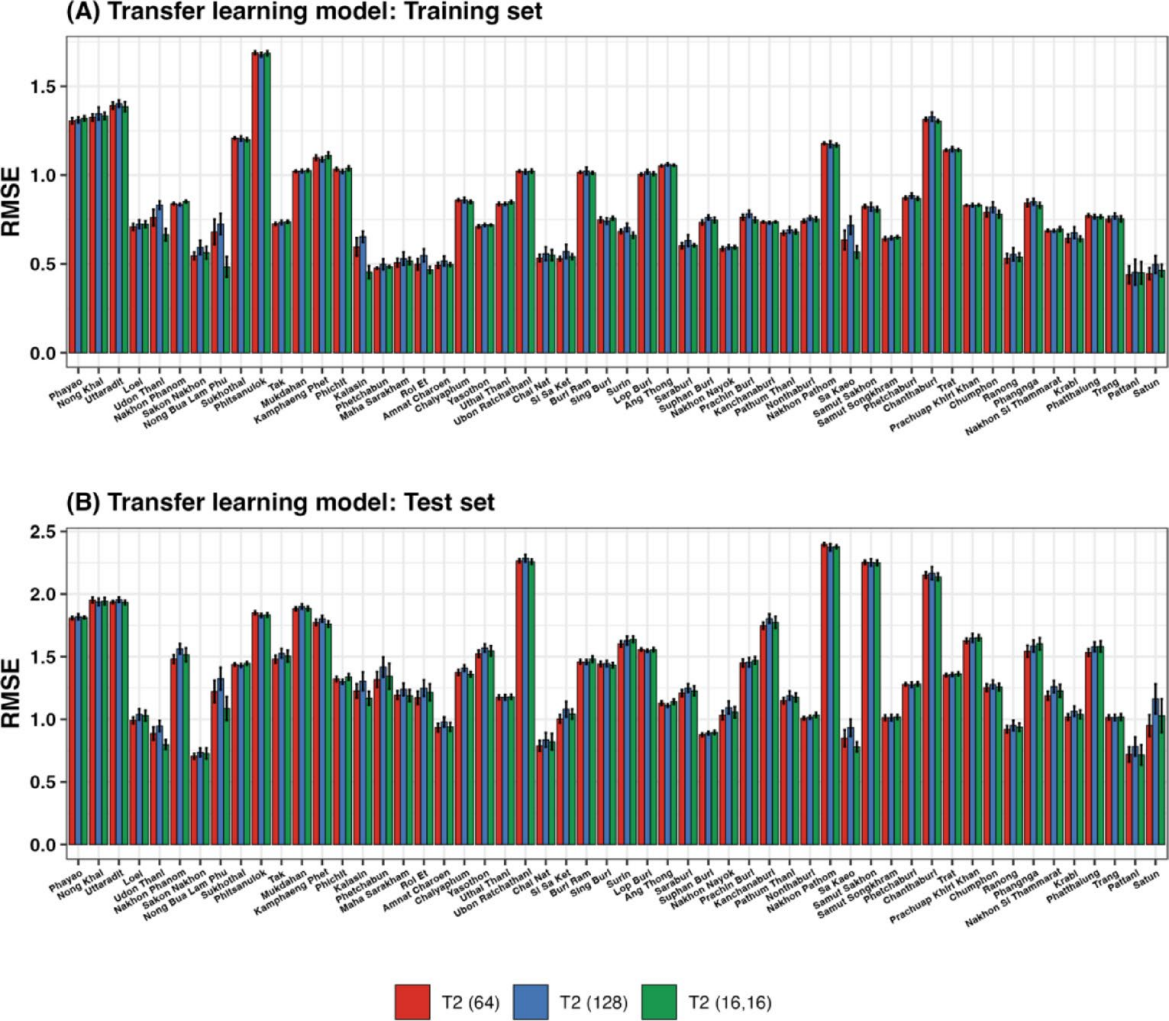
Average RMSE values for the training (**A**) and testing (**B**) sets of the T2 models over 50 runs. The bars indicate the mean RMSE values, and the error bars indicate the 95% confidence intervals.

The RMSE (and MAE) values of the T2 models for each province are presented in Fig. 7 (and Figure S15). Most provinces exhibited relatively low RMSE values in the training set, indicating that the models performed well during this phase (Fig. 7A). However, some provinces displayed significantly higher RMSE values. In the test set (Fig. 7B), the RMSE values are mostly higher than those in the training set, which is expected, as the models typically perform better on training data than on unseen data. Similar to the training results, some provinces exhibited very high RMSE values, indicating that the models had difficulty making accurate predictions in these areas.

**Fig. 8:**
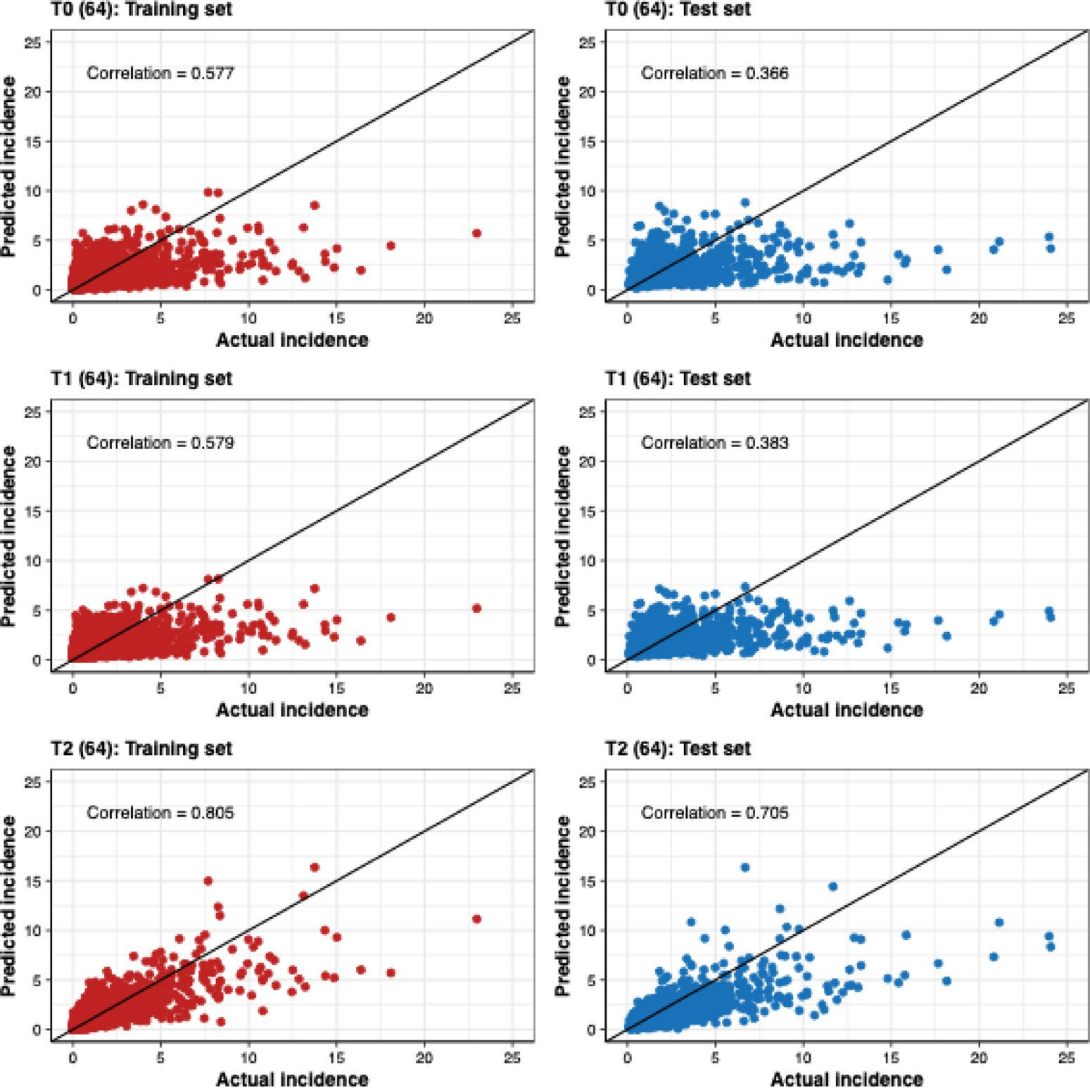
Scatter plots comparing the predicted incidence with the actual incidence for the training and testing sets using the T2 model with 64 hidden units.

Figure 8 displays scatter plots comparing actual incidence with predicted incidence for both the training and test sets using the models with 64 hidden units. The diagonal line represents the line of equality ($\:y\:=\:x$) to confirm that the T2 model with 64 hidden units outperforms the T0 and T1 models, with more data points ($\:x$, $\:y$) clustering closer to this diagonal line. Overall, T2 shows the strongest association with observations, with higher correlations in both the training set ($\:r$ = 0.805) and the test set ($\:r$ = 0.705), compared with T0 ($\:r$ = 0.577 train; $\:r$ = 0.366 test) and T1 ($\:r$ = 0.579 train; $\:r$ = 0.383 test). These correlation patterns indicate that the T2 model with 64 hidden units demonstrated superior performance on both training and testing sets, highlighting the benefits of province-specific fine-tuning. Despite this improvement, the scatter plots also suggest systematic underestimation at higher incidence levels. For large observed values, many points fall below the equality line, indicating that the model tends to under-fit peak magnitudes. This under-forecasting is most evident in the test set, where extreme peaks are hard to learn.

## Discussion

In this study, we presented a universal ANN model for predicting influenza incidence. Most existing models are specific to particular areas, requiring separate training for each location. This area-specific approach complicates the efforts of public health officials and policymakers. It is challenging to construct a universal model suitable for multiple areas. We developed a universal model using data from 22 provinces across different regions of Thailand from 2010 to 2019. To identify the best model, we compared the performance of models with several configurations. We also employed a feature selection method to ensure that features from multiple time series and different provinces contributed equally to the model, thereby improving its generalizability and performance. This approach helps reduce spatial and temporal biases, making the universal model more robust. Furthermore, reducing the number of input features for training ANN models improves computational efficiency and reduces training time.

 Transfer learning offers better performance and efficiency by utilizing a model trained on one task and applying it to another related task^19,20,34^. This technique is particularly advantageous when the new task has limited data. Specifically, after training on a similar domain dataset, the model becomes a pre-trained model. This pre-trained model is then fine-tuned using the target dataset, enhancing its generalizability and making it applicable to a wider range of datasets.

To our knowledge, this is the first study predicting influenza incidence in Thailand using transfer learning with a large dataset. We compared three different models (T0, T1, and T2) and developed a transfer learning approach based on a universal ANN framework. Our transfer learning models with selected features (T1 and T2) outperformed the universal ANN model without transfer learning (T0). However, while T1 outperformed T0, it still underperformed compared to the T2 model. The T1 model may not fit well due to the lack of province-specific fine-tuning, which could have led to less accurate adjustments for different areas. This suggests that fine-tuning models for specific provinces, as in the T2 approach, provides additional flexibility in adapting to local data variations. However, we assumed that influenza incidence in Thailand forms a homogeneous system, meaning that features from one province can be used to train the model and predict incidence rates in other provinces. When applying this model to other regions, it is important to consider diverse climates and landscapes.

We also investigated the impact of meteorological and PM10 features by comparing model performance. The model incorporated various meteorological factors, including temperature, relative humidity, precipitation, wind speed, and visibility, for predictions. PM10 concentration was also included, as recursive feature selection identified it as an important predictor. However, meteorological factors may have different impacts depending on the region. Our findings revealed that the universal model using meteorological features (M2) did not enhance overall performance. Improvements were only observed in specific provinces, such as Chiang Mai, Nan, and Nakhon Sawan, where the M2 model aligned more closely with the actual data. This suggests that meteorological data may significantly influence the incidence rate in certain provinces^21^. Nevertheless, results from the transfer learning models indicated that incorporating these features improved overall performance. Notably, temperature was selected as a feature across all 50 runs for all 22 provinces, suggesting that it plays an important role in influenza incidence, consistent with previous studies^19,35^. Further investigation is necessary to explore how meteorological and PM10 factors influence incidence rates at the provincial level.

Our model effectively captures the spatial and temporal relationships of incidence rates across 22 provinces and successfully transfers this information to 54 other provinces using transfer learning models. Our experiments demonstrated that TL models with a single hidden layer outperformed those with double hidden layers, with 64 hidden units yielding the best performance. This suggests that increasing model complexity (i.e., adding more hidden layers and nodes) did not improve performance. This may be due to the limited size of the dataset, which lacks sufficient sequences to accurately capture temporal patterns. With such a dataset, a more complex model can lead to low bias but high variance, as noted in^36^. An increase in nodes significantly raises the number of parameters, further complicating the model. Therefore, a simpler model proved to be a better choice for this study. Similar findings were observed in previous studies, such as the evaluation of pneumoconiosis, where a Deep Neural Network model demonstrated superior generalization ability^37^.

We observed systematic underestimation of incidence in several provinces (e.g., Bangkok, Rayong, Chiang Mai, and Lampang; Fig. 5 and Figure S13), and a similar pattern is evident in the TL results (Fig. 8), where predictions at higher observed incidence often fall below the line of equality. This under-forecasting may be due to the universal and TL models were optimized to minimize average error across a large number of province–month observations. Since low-to-moderate incidences are much more frequent than extreme peaks, the fitted models tend to produce smoother predictions that shrink extreme values toward the overall mean. Consequently, while the models capture temporal co-movement reasonably well (e.g., for T2(64), correlations of 0.805 in training and 0.705 in the test set), they may underestimate the magnitude of large outbreaks and thus should be interpreted primarily as tools for forecasting overall trends and relative changes rather than the absolute height of extreme peaks.

Our study has some limitations. We did not account for other factors, such as economic conditions^38,39^ and mobility patterns^39^, which may influence influenza incidence. Additionally, we did not explore hybrid models that combine fractal theory and fuzzy logic^40^, which have shown potential for improving model accuracy in COVID-19 predictions. Our focus, however, was on basic deep learning models to simplify their application in public health. Furthermore, our model is designed to predict incidence rates for a single time step, while multi-step-ahead predictions could be more beneficial for early warning systems. However, implementing such predictions may introduce additional errors and increase model complexity.

## Conclusion

We developed a universal ANN model to predict influenza incidence across multiple provinces in Thailand, using data from 22 provinces as a case study. Our models demonstrated strong potential for accurate predictions, with transfer learning (TL) enhancing the accuracy of predictions in provinces where feature data are unavailable. Among the TL models, the T2 model with 64 hidden units achieved the lowest average RMSE in most provinces, demonstrating effective generalization. These findings highlight the value of TL in leveraging knowledge from data-rich provinces to support early warning systems and resource allocation. However, this study did not account for socioeconomic and mobility factors, which may also influence influenza incidence. Future research could explore hybrid models and multi-step-ahead forecasts to improve prediction accuracy and strengthen disease management strategies.

## Supplementary Information

Below is the link to the electronic supplementary material.

Supplementary Material 1

## Data Availability

Data is provided within the manuscript or supplementary information files.

## References

[1] Lafond, K. E. et al. Global burden of influenza-associated lower respiratory tract infections and hospitalizations among adults: A systematic review and meta-analysis. *PLoS Med.* **18**, e1003550 (2021). 33647033 10.1371/journal.pmed.1003550 PMC7959367

[2] Dawood, F. S. et al. Estimated global mortality associated with the first 12 months of 2009 pandemic influenza A H1N1 virus circulation: a modelling study. *Lancet Infect. Dis.* **12**, 687–695 (2012). 22738893 10.1016/S1473-3099(12)70121-4

[3] Jones, N. Why easing COVID restrictions could prompt a fierce flu rebound. *Nature* **598**, 395 (2021). 34621039 10.1038/d41586-021-02558-8

[4] Centers for Disease Control and Prevention. Past Seasons’ Flu Season Severity Assessments. https://www.cdc.gov/flu/about/classifies-flu-severity.htm (2024).

[5] Suntronwong, N. et al. Climate factors influence seasonal influenza activity in Bangkok, Thailand. *PLoS One*. **15**, e0239729 (2020). 32991630 10.1371/journal.pone.0239729 PMC7523966

[6] Chadsuthi, S., Iamsirithaworn, S., Triampo, W. & Modchang, C. Modeling Seasonal Influenza Transmission and Its Association with Climate Factors in Thailand Using Time-Series and ARIMAX Analyses. *Comput. Math. Methods Med.* (2015). (2015). 10.1155/2015/436495 PMC4667155 26664492

[7] Jainonthee, C., Wang, Y. L., Chen, C. W. K. & Jainontee, K. Air Pollution-Related respiratory diseases and associated environmental factors in Chiang Mai, Thailand, in 2011–2020. *Trop. Med. Infect. Dis.* **7**, (2022). 10.3390/tropicalmed7110341 PMC9696662 36355883

[8] Nsoesie, E. O., Brownstein, J. S., Ramakrishnan, N. & Marathe, M. V. A systematic review of studies on forecasting the dynamics of influenza outbreaks. *Influenza Other Respir Viruses*. **8**, 309–316 (2014). 24373466 10.1111/irv.12226 PMC4181479

[9] Wang, X. L. et al. Model selection in time series studies of influenza-associated mortality. *PLoS One*. **7**, e39423 (2012). 22745751 10.1371/journal.pone.0039423 PMC3380027

[10] Santangelo, O. E., Gentile, V., Pizzo, S., Giordano, D. & Cedrone, F. Machine learning and prediction of infectious diseases: A systematic review. *Mach. Learn. Knowl. Extr.* **5**, 175–198 (2023).

[11] Lee, T. S., Chen, I. F., Chang, T. J. & Lu, C. J. Forecasting weekly influenza outpatient visits using a Two-Dimensional hierarchical decision tree scheme. *Int. J. Environ. Res. Public. Health*. **17**, 4743 (2020). 32630311 10.3390/ijerph17134743 PMC7369891

[12] Hu, H. et al. Prediction of influenza-like illness based on the improved artificial tree algorithm and artificial neural network. *Sci. Rep.* **8**, 4895 (2018). 29559649 10.1038/s41598-018-23075-1 PMC5861130

[13] Nikparvar, B., Rahman, M. M., Hatami, F. & Thill, J. C. Spatio-temporal prediction of the COVID-19 pandemic in US counties: modeling with a deep LSTM neural network. *Sci. Rep.* **11**, 21715 (2021). 34741093 10.1038/s41598-021-01119-3 PMC8571358

[14] Winalai, C., Anupong, S., Modchang, C., Chadsuthi, S. & LSTM-Powered COVID-19 prediction in central Thailand incorporating meteorological and particulate matter data with a multi-feature selection approach. *Heliyon* **10**, (2024). 10.1016/j.heliyon.2024.e30319 PMC11070856 38711630

[15] Kwofie, S. K. et al. Artificial Intelligence, Machine Learning, and Big Data for Ebola Virus Drug Discovery. Pharmaceuticals vol. 16 10.3390/ph16030332 (2023). 10.3390/ph16030332 PMC10052301 36986432

[16] Azeem, M. et al. Neural networks for the detection of COVID-19 and other diseases: prospects and challenges. *Bioengineering (Basel)* **10**, (2023). 10.3390/bioengineering10070850 PMC10416184 37508877

[17] Alnaji, L. Machine learning in epidemiology: neural networks forecasting of Monkeypox cases. *PLoS One*. **19**, e0300216 (2024). 38691574 10.1371/journal.pone.0300216 PMC11062558

[18] Zan, A. et al. DeepFlu: a deep learning approach for forecasting symptomatic influenza A infection based on pre-exposure gene expression: forecasting symptomatic influenza A infection by deepflu. *Comput. Methods Programs Biomed.* **213**, (2022). 10.1016/j.cmpb.2021.106495 34798406

[19] Xue, D., Wang, M., Liu, F. & Buss, M. Time series modeling and forecasting of epidemic spreading processes using deep transfer learning. *Chaos Solitons Fractals*. **185**, 115092 (2024).

[20] Roster, K., Connaughton, C. & Rodrigues, F. A. Forecasting new diseases in low-data settings using transfer learning. *Chaos Solitons Fractals*. **161**, 112306 (2022). 35765601 10.1016/j.chaos.2022.112306 PMC9222348

[21] Anupong, S., Modchang, C. & Chadsuthi, S. Seasonal patterns of influenza incidence and the influence of meteorological and air pollution factors in Thailand during 2009–2019. *Heliyon* **10**, e36703 (2024). 39263141 10.1016/j.heliyon.2024.e36703 PMC11388739

[22] Division of Epidemiology, Department of Disease Control & Ministry of Public Health. Case Definition for Communicable Diseases Surveillance. (Bangkok, Thailand, (2020).

[23] Sparks, A. H., Hengl, T. & Nelson, A. GSODR: global summary daily weather data in R. *J. Open. Source Softw.* **2**, 177 (2017).

[24] Pollution Control Department & Ministry of Natural Resources and Environment. Air4Thai. http://air4thai.pcd.go.th/webV3/#/Home (2023).

[25] Breiman, L. Random forests. *Mach. Learn.* **45**, 5–32 (2001).

[26] Pires, P. B., Santos, J. D. & Pereira, I. V. Artificial neural networks: history and state of the Art. *Encyclopedia Inform. Sci. Technol. Sixth Ed.* 1–25 (2025).

[27] Pointer, I. *Programming PyTorch for Deep Learning: Creating and Deploying Deep Learning Applications* (O’Reilly Media, Inc, 2019).

[28] Kingma Ba, J. & Adam, P. D. A Method for Stochastic Optimization. CoRR https://api.semanticscholar.org/CorpusID:6628106 (2014).

[29] R Core Team. R: A Language and Environment for Statistical Computing. https://www.R-project.org (2022).

[30] Hadley, W. et al. Welcome to the tidyverse. *J. Open. Source Softw.* **4**, 1686 (2019).

[31] Alboukadel, K. & ggpubr ‘ggplot2’ Based Publication Ready Plots. https://CRAN.R-project.org/package=ggpubr. (2020).

[32] Bivand, R. S. & Wong, D. W. S. Comparing implementations of global and local indicators of Spatial association. *Test* **27**, 716–748 (2018).

[33] Pebesma, E. J. Simple features for R: standardized support for Spatial vector data. *R J.* **10**, 439 (2018).

[34] Ye, R. & Dai, Q. A novel transfer learning framework for time series forecasting. *Knowl. Based Syst.* **156**, 74–99 (2018).

[35] Zheng, Y., Wang, K., Zhang, L. & Wang, L. Study on the relationship between the incidence of influenza and climate indicators and the prediction of influenza incidence. *Environ. Sci. Pollut. Res.* **28**, 473–481 (2021). 10.1007/s11356-020-10523-7 32815008

[36] Lever, J., Krzywinski, M. & Altman, N. Model selection and overfitting. *Nat. Methods*. **13**, 703–704 (2016).

[37] Lou, H. R., Wang, X., Gao, Y. & Zeng, Q. Comparison of ARIMA model, DNN model and LSTM model in predicting disease burden of occupational pneumoconiosis in Tianjin, China. *BMC Public. Health*. **22**, 2167 (2022). 36434563 10.1186/s12889-022-14642-3 PMC9694549

[38] Prager, F., Wei, D. & Rose, A. Total economic consequences of an influenza outbreak in the united States. *Risk Anal.* **37**, 4–19 (2017). 27214756 10.1111/risa.12625

[39] Burris, C., Nikolaev, A., Zhong, S. & Bian, L. Network effects in influenza spread: the impact of mobility and socio-economic factors. *Socioecon Plann. Sci.* **78**, 101081 (2021). 35812715 10.1016/j.seps.2021.101081 PMC9264374

[40] Castillo, O. & Melin, P. Forecasting of COVID-19 time series for countries in the world based on a hybrid approach combining the fractal dimension and fuzzy logic. *Chaos Solitons Fractals*. **140**, 110242 (2020). 32863616 10.1016/j.chaos.2020.110242 PMC7444908

