# Supplementary material for: Leveraging universal and transfer learning models for influenza prediction in Thailand
Source: Sci Rep. 2026 Jan 30;16:6668. doi: 10.1038/s41598-026-37855-7 (PMC12913657; doi:10.1038/s41598-026-37855-7)
Supplement: Supplementary file 1 — Supplementary Material 1 [file 41598_2026_37855_MOESM1_ESM.docx]

**Supplementary Materials**

**Table S1** The average of RMSE of training set for universal model.

| **Province** | **LR_M1** | **LR_M2** | **XGBoost_M1** | **XGBoost_M2** | **ANN_M1 (8)** | **ANN_M2 (8)** | **ANN_M1 (16)** | **ANN_M2 (16)** | **ANN_M1 (32)** | **ANN_M2 (32)** | **ANN_M1 (64)** | **ANN_M2 (64)** | **ANN_M1 (128)** | **ANN_M2 (128)** | **ANN_M1 (8,8)** | **ANN_M2 (8,8)** | **ANN_M1 (16,16)** | **ANN_M2 (16,16)** | **ANN_M1 (32,32)** | **ANN_M2 (32,32)** |
| --- | --- | --- | --- | --- | --- | --- | --- | --- | --- | --- | --- | --- | --- | --- | --- | --- | --- | --- | --- | --- |
| **Chiang Rai** | 1.014 | 0.986 | 0.219 | 0.097 | 0.985 | 0.976 | 0.987 | 0.978 | 0.987 | 0.983 | 0.991 | 0.989 | 1.001 | 1.000 | 1.036 | 1.024 | 1.023 | 1.012 | 1.056 | 1.031 |
| **Mae Hong Son** | 1.307 | 1.303 | 0.259 | 0.171 | 1.062 | 1.059 | 1.068 | 1.072 | 1.069 | 1.073 | 1.074 | 1.075 | 1.093 | 1.086 | 1.076 | 1.081 | 1.073 | 1.084 | 1.090 | 1.097 |
| **Chiang Mai** | 2.544 | 2.535 | 0.328 | 0.189 | 1.694 | 1.692 | 1.686 | 1.681 | 1.680 | 1.675 | 1.669 | 1.670 | 1.638 | 1.652 | 1.710 | 1.708 | 1.690 | 1.682 | 1.679 | 1.674 |
| **Nan** | 1.102 | 1.092 | 0.256 | 0.152 | 1.054 | 1.053 | 1.042 | 1.044 | 1.035 | 1.039 | 1.037 | 1.034 | 1.048 | 1.038 | 1.071 | 1.070 | 1.035 | 1.043 | 1.051 | 1.048 |
| **Lamphun** | 1.571 | 1.540 | 0.296 | 0.168 | 1.293 | 1.285 | 1.286 | 1.279 | 1.283 | 1.275 | 1.278 | 1.275 | 1.264 | 1.268 | 1.319 | 1.311 | 1.297 | 1.288 | 1.296 | 1.287 |
| **Lampang** | 2.700 | 2.672 | 0.388 | 0.187 | 1.703 | 1.697 | 1.696 | 1.688 | 1.689 | 1.682 | 1.672 | 1.679 | 1.633 | 1.655 | 1.719 | 1.720 | 1.702 | 1.696 | 1.696 | 1.689 |
| **Phrae** | 1.604 | 1.604 | 0.301 | 0.185 | 1.165 | 1.167 | 1.174 | 1.178 | 1.181 | 1.181 | 1.182 | 1.187 | 1.182 | 1.182 | 1.188 | 1.186 | 1.200 | 1.195 | 1.221 | 1.209 |
| **Khon Kaen** | 1.003 | 1.015 | 0.222 | 0.155 | 0.905 | 0.899 | 0.903 | 0.912 | 0.899 | 0.908 | 0.906 | 0.907 | 0.925 | 0.927 | 0.875 | 0.893 | 0.846 | 0.898 | 0.871 | 0.903 |
| **Nakhon Sawan** | 1.167 | 1.140 | 0.259 | 0.152 | 1.138 | 1.134 | 1.128 | 1.122 | 1.115 | 1.113 | 1.104 | 1.104 | 1.062 | 1.078 | 1.119 | 1.129 | 1.102 | 1.124 | 1.108 | 1.112 |
| **Nakhon Ratchasima** | 3.397 | 3.380 | 0.462 | 0.231 | 1.339 | 1.338 | 1.325 | 1.320 | 1.313 | 1.309 | 1.306 | 1.298 | 1.280 | 1.276 | 1.333 | 1.336 | 1.306 | 1.311 | 1.300 | 1.295 |
| **Phra Nakhon Si Ayutthaya** | 1.230 | 1.204 | 0.179 | 0.135 | 1.133 | 1.124 | 1.127 | 1.115 | 1.114 | 1.113 | 1.113 | 1.112 | 1.103 | 1.108 | 1.163 | 1.156 | 1.138 | 1.139 | 1.161 | 1.142 |
| **Bangkok** | 3.811 | 3.779 | 0.489 | 0.274 | 2.026 | 2.024 | 2.017 | 2.011 | 2.009 | 2.004 | 2.000 | 1.997 | 1.972 | 1.978 | 2.039 | 2.038 | 2.015 | 2.013 | 2.004 | 2.002 |
| **Chachoengsao** | 0.953 | 0.962 | 0.218 | 0.167 | 0.977 | 0.980 | 0.969 | 0.977 | 0.959 | 0.966 | 0.949 | 0.957 | 0.914 | 0.940 | 0.929 | 0.954 | 0.908 | 0.958 | 0.921 | 0.952 |
| **Samut Prakan** | 1.089 | 1.079 | 0.207 | 0.133 | 1.103 | 1.094 | 1.098 | 1.088 | 1.087 | 1.082 | 1.079 | 1.078 | 1.057 | 1.063 | 1.126 | 1.131 | 1.116 | 1.120 | 1.126 | 1.121 |
| **Ratchaburi** | 1.211 | 1.227 | 0.241 | 0.124 | 1.019 | 1.020 | 1.030 | 1.032 | 1.039 | 1.036 | 1.041 | 1.043 | 1.043 | 1.040 | 1.067 | 1.071 | 1.067 | 1.077 | 1.096 | 1.094 |
| **Chon Buri** | 1.228 | 1.234 | 0.261 | 0.137 | 1.080 | 1.078 | 1.092 | 1.087 | 1.089 | 1.089 | 1.088 | 1.090 | 1.087 | 1.081 | 1.109 | 1.115 | 1.121 | 1.126 | 1.147 | 1.138 |
| **Rayong** | 3.484 | 3.460 | 0.448 | 0.238 | 1.937 | 1.935 | 1.927 | 1.921 | 1.919 | 1.913 | 1.908 | 1.907 | 1.878 | 1.885 | 1.952 | 1.951 | 1.926 | 1.924 | 1.912 | 1.912 |
| **Surat Thani** | 0.822 | 0.817 | 0.248 | 0.128 | 0.934 | 0.923 | 0.928 | 0.915 | 0.921 | 0.913 | 0.920 | 0.911 | 0.921 | 0.907 | 0.971 | 0.963 | 0.949 | 0.943 | 0.977 | 0.955 |
| **Phuket** | 1.443 | 1.418 | 0.278 | 0.165 | 1.224 | 1.224 | 1.224 | 1.218 | 1.220 | 1.221 | 1.218 | 1.224 | 1.213 | 1.218 | 1.279 | 1.274 | 1.261 | 1.251 | 1.268 | 1.260 |
| **Songkhla** | 1.608 | 1.619 | 0.241 | 0.136 | 1.099 | 1.087 | 1.110 | 1.104 | 1.115 | 1.107 | 1.128 | 1.114 | 1.150 | 1.120 | 1.133 | 1.119 | 1.145 | 1.144 | 1.190 | 1.156 |
| **Yala** | 1.698 | 1.692 | 0.271 | 0.147 | 1.168 | 1.160 | 1.177 | 1.174 | 1.185 | 1.178 | 1.198 | 1.183 | 1.228 | 1.188 | 1.167 | 1.167 | 1.195 | 1.190 | 1.223 | 1.204 |
| **Narathiwat** | 1.383 | 1.405 | 0.255 | 0.143 | 1.037 | 1.030 | 1.043 | 1.041 | 1.049 | 1.043 | 1.059 | 1.048 | 1.082 | 1.048 | 1.047 | 1.043 | 1.051 | 1.054 | 1.084 | 1.066 |

M1 = The model that use only previous incidence for feature selection.

M2 = The model that use previous incidence, meteorological, and particulate matter for feature selection.

(node)= The number of nodes of 1 layer. (node, node) = The number of nodes of 2 layer.

**Table S2** The average of RMSE of test set for universal model.

| **Province** | **LR_M1** | **LR_M2** | **XGBoost_M1** | **XGBoost_M2** | **ANN_M1 (8)** | **ANN_M2 (8)** | **ANN_M1 (16)** | **ANN_M2 (16)** | **ANN_M1 (32)** | **ANN_M2 (32)** | **ANN_M1 (64)** | **ANN_M2 (64)** | **ANN_M1 (128)** | **ANN_M2 (128)** | **ANN_M1 (8,8)** | **ANN_M2 (8,8)** | **ANN_M1 (16,16)** | **ANN_M2 (16,16)** | **ANN_M1 (32,32)** | **ANN_M2 (32,32)** |
| --- | --- | --- | --- | --- | --- | --- | --- | --- | --- | --- | --- | --- | --- | --- | --- | --- | --- | --- | --- | --- |
| **Chiang Rai** | 2.182 | 2.100 | 2.255 | 2.765 | 1.538 | 1.531 | 1.546 | 1.532 | 1.541 | 1.540 | 1.537 | 1.551 | 1.516 | 1.569 | 1.606 | 1.586 | 1.602 | 1.588 | 1.648 | 1.618 |
| **Mae Hong Son** | 2.475 | 2.384 | 2.534 | 3.650 | 1.543 | 1.523 | 1.577 | 1.549 | 1.579 | 1.574 | 1.574 | 1.597 | 1.550 | 1.589 | 1.635 | 1.621 | 1.675 | 1.667 | 1.754 | 1.707 |
| **Chiang Mai** | 7.424 | 7.441 | 9.341 | 8.959 | 2.897 | 2.903 | 2.878 | 2.885 | 2.857 | 2.873 | 2.855 | 2.858 | 2.838 | 2.867 | 2.857 | 2.868 | 2.816 | 2.833 | 2.797 | 2.814 |
| **Nan** | 2.226 | 2.176 | 3.326 | 3.073 | 1.671 | 1.680 | 1.658 | 1.658 | 1.645 | 1.658 | 1.634 | 1.651 | 1.606 | 1.645 | 1.664 | 1.679 | 1.633 | 1.641 | 1.647 | 1.649 |
| **Lamphun** | 3.313 | 3.311 | 4.498 | 5.215 | 1.988 | 1.993 | 1.961 | 1.973 | 1.940 | 1.956 | 1.936 | 1.943 | 1.906 | 1.965 | 1.977 | 1.968 | 1.915 | 1.926 | 1.891 | 1.904 |
| **Lampang** | 3.556 | 3.554 | 3.355 | 3.631 | 2.012 | 2.016 | 2.003 | 1.999 | 1.993 | 1.994 | 1.986 | 1.986 | 1.970 | 1.970 | 2.004 | 2.015 | 1.973 | 1.981 | 1.963 | 1.974 |
| **Phrae** | 3.327 | 3.243 | 3.652 | 3.845 | 1.612 | 1.599 | 1.638 | 1.619 | 1.674 | 1.638 | 1.667 | 1.671 | 1.639 | 1.654 | 1.809 | 1.754 | 1.800 | 1.766 | 1.866 | 1.801 |
| **Khon Kaen** | 3.048 | 2.936 | 2.887 | 3.552 | 1.553 | 1.541 | 1.586 | 1.569 | 1.613 | 1.599 | 1.614 | 1.635 | 1.610 | 1.646 | 1.729 | 1.685 | 1.736 | 1.716 | 1.813 | 1.770 |
| **Nakhon Sawan** | 3.209 | 3.223 | 4.232 | 3.982 | 1.977 | 1.992 | 1.947 | 1.956 | 1.928 | 1.940 | 1.914 | 1.918 | 1.896 | 1.888 | 1.935 | 1.958 | 1.873 | 1.885 | 1.848 | 1.867 |
| **Nakhon Ratchasima** | 9.732 | 9.836 | 10.424 | 9.993 | 2.295 | 2.314 | 2.270 | 2.281 | 2.247 | 2.268 | 2.235 | 2.246 | 2.208 | 2.224 | 2.231 | 2.270 | 2.186 | 2.211 | 2.164 | 2.192 |
| **Phra Nakhon Si Ayutthaya** | 2.093 | 2.061 | 3.131 | 3.265 | 1.460 | 1.454 | 1.460 | 1.452 | 1.452 | 1.455 | 1.446 | 1.460 | 1.432 | 1.462 | 1.500 | 1.505 | 1.492 | 1.504 | 1.515 | 1.518 |
| **Bangkok** | 9.785 | 9.803 | 10.057 | 9.661 | 3.274 | 3.281 | 3.253 | 3.255 | 3.241 | 3.244 | 3.234 | 3.228 | 3.222 | 3.206 | 3.249 | 3.262 | 3.202 | 3.207 | 3.177 | 3.188 |
| **Chachoengsao** | 2.780 | 2.798 | 2.762 | 3.145 | 1.885 | 1.902 | 1.852 | 1.861 | 1.826 | 1.845 | 1.812 | 1.822 | 1.804 | 1.791 | 1.827 | 1.867 | 1.772 | 1.793 | 1.771 | 1.787 |
| **Samut Prakan** | 2.083 | 2.040 | 2.630 | 3.188 | 1.619 | 1.620 | 1.600 | 1.591 | 1.584 | 1.584 | 1.570 | 1.575 | 1.530 | 1.541 | 1.620 | 1.628 | 1.582 | 1.584 | 1.594 | 1.583 |
| **Ratchaburi** | 2.243 | 2.181 | 3.370 | 3.529 | 1.322 | 1.308 | 1.299 | 1.297 | 1.305 | 1.298 | 1.297 | 1.318 | 1.300 | 1.333 | 1.461 | 1.418 | 1.399 | 1.392 | 1.462 | 1.430 |
| **Chon Buri** | 1.464 | 1.408 | 2.172 | 2.123 | 1.331 | 1.344 | 1.289 | 1.296 | 1.262 | 1.281 | 1.249 | 1.267 | 1.246 | 1.246 | 1.312 | 1.338 | 1.255 | 1.274 | 1.300 | 1.291 |
| **Rayong** | 8.496 | 8.581 | 9.138 | 8.952 | 3.080 | 3.093 | 3.053 | 3.062 | 3.035 | 3.048 | 3.023 | 3.028 | 3.006 | 3.005 | 3.032 | 3.063 | 2.983 | 3.002 | 2.953 | 2.983 |
| **Surat Thani** | 1.701 | 1.667 | 2.480 | 2.393 | 1.419 | 1.422 | 1.400 | 1.399 | 1.386 | 1.398 | 1.379 | 1.397 | 1.343 | 1.402 | 1.463 | 1.450 | 1.418 | 1.422 | 1.456 | 1.431 |
| **Phuket** | 3.501 | 3.509 | 5.039 | 4.282 | 1.987 | 1.998 | 1.952 | 1.962 | 1.940 | 1.948 | 1.925 | 1.932 | 1.927 | 1.915 | 1.994 | 2.007 | 1.909 | 1.917 | 1.882 | 1.912 |
| **Songkhla** | 3.136 | 3.160 | 4.055 | 1.689 | 1.475 | 1.467 | 1.534 | 1.518 | 1.573 | 1.549 | 1.585 | 1.586 | 1.583 | 1.567 | 1.653 | 1.620 | 1.704 | 1.667 | 1.774 | 1.702 |
| **Yala** | 3.874 | 3.893 | 4.462 | 2.385 | 1.581 | 1.563 | 1.645 | 1.623 | 1.689 | 1.663 | 1.712 | 1.709 | 1.722 | 1.710 | 1.791 | 1.741 | 1.856 | 1.822 | 1.947 | 1.863 |
| **Narathiwat** | 3.228 | 3.183 | 3.368 | 2.168 | 1.406 | 1.375 | 1.480 | 1.446 | 1.516 | 1.487 | 1.536 | 1.537 | 1.545 | 1.545 | 1.640 | 1.589 | 1.679 | 1.662 | 1.789 | 1.714 |

M1 = The model that use only previous incidence for feature selection.

M2 = The model that use previous incidence, meteorological, and particulate matter for feature selection.

(node)= The number of nodes of 1 layer. (node, node) = The number of nodes of 2 layer.

**Table S3** The average of MAE of training set for universal model.

| **Province** | **LR_M1** | **LR_M2** | **XGBoost_M1** | **XGBoost_M2** | **ANN_M1 (8)** | **ANN_M2 (8)** | **ANN_M1 (16)** | **ANN_M2 (16)** | **ANN_M1 (32)** | **ANN_M2 (32)** | **ANN_M1 (64)** | **ANN_M2 (64)** | **ANN_M1 (128)** | **ANN_M2 (128)** | **ANN_M1 (8,8)** | **ANN_M2 (8,8)** | **ANN_M1 (16,16)** | **ANN_M2 (16,16)** | **ANN_M1 (32,32)** | **ANN_M2 (32,32)** |
| --- | --- | --- | --- | --- | --- | --- | --- | --- | --- | --- | --- | --- | --- | --- | --- | --- | --- | --- | --- | --- |
| **Chiang Rai** | 0.685 | 0.669 | 0.167 | 0.076 | 0.683 | 0.667 | 0.677 | 0.668 | 0.670 | 0.671 | 0.671 | 0.669 | 0.662 | 0.670 | 0.740 | 0.729 | 0.710 | 0.698 | 0.766 | 0.713 |
| **Mae Hong Son** | 1.049 | 1.051 | 0.187 | 0.121 | 0.899 | 0.894 | 0.898 | 0.912 | 0.899 | 0.901 | 0.899 | 0.899 | 0.899 | 0.902 | 0.818 | 0.857 | 0.793 | 0.858 | 0.795 | 0.850 |
| **Chiang Mai** | 1.567 | 1.567 | 0.231 | 0.130 | 1.832 | 1.836 | 1.808 | 1.805 | 1.788 | 1.791 | 1.767 | 1.778 | 1.697 | 1.734 | 1.917 | 1.922 | 1.850 | 1.845 | 1.837 | 1.832 |
| **Nan** | 0.786 | 0.768 | 0.198 | 0.106 | 0.770 | 0.764 | 0.743 | 0.749 | 0.728 | 0.730 | 0.722 | 0.713 | 0.718 | 0.709 | 0.737 | 0.758 | 0.678 | 0.714 | 0.707 | 0.716 |
| **Lamphun** | 0.977 | 0.972 | 0.210 | 0.111 | 1.056 | 1.035 | 1.043 | 1.025 | 1.031 | 1.013 | 1.025 | 1.004 | 0.983 | 0.987 | 1.076 | 1.065 | 1.039 | 1.032 | 1.051 | 1.013 |
| **Lampang** | 1.695 | 1.675 | 0.253 | 0.125 | 1.875 | 1.872 | 1.864 | 1.851 | 1.846 | 1.848 | 1.820 | 1.845 | 1.753 | 1.804 | 1.986 | 1.985 | 1.925 | 1.893 | 1.922 | 1.899 |
| **Phrae** | 1.220 | 1.221 | 0.240 | 0.146 | 1.023 | 1.011 | 1.020 | 1.029 | 1.016 | 1.015 | 1.011 | 1.009 | 0.995 | 1.004 | 0.855 | 0.900 | 0.819 | 0.899 | 0.807 | 0.882 |
| **Khon Kaen** | 0.858 | 0.864 | 0.185 | 0.113 | 0.716 | 0.704 | 0.708 | 0.721 | 0.703 | 0.708 | 0.699 | 0.701 | 0.685 | 0.706 | 0.602 | 0.650 | 0.562 | 0.653 | 0.573 | 0.641 |
| **Nakhon Sawan** | 0.818 | 0.799 | 0.183 | 0.106 | 0.901 | 0.889 | 0.878 | 0.876 | 0.856 | 0.853 | 0.834 | 0.837 | 0.762 | 0.800 | 0.796 | 0.834 | 0.764 | 0.834 | 0.791 | 0.811 |
| **Nakhon Ratchasima** | 1.738 | 1.739 | 0.325 | 0.159 | 1.094 | 1.086 | 1.069 | 1.061 | 1.049 | 1.041 | 1.032 | 1.025 | 0.978 | 0.988 | 1.047 | 1.077 | 1.003 | 1.048 | 1.014 | 1.030 |
| **Phra Nakhon Si Ayutthaya** | 0.958 | 0.936 | 0.122 | 0.092 | 0.938 | 0.921 | 0.934 | 0.911 | 0.907 | 0.911 | 0.917 | 0.911 | 0.909 | 0.916 | 1.038 | 1.022 | 0.973 | 0.969 | 1.027 | 0.974 |
| **Bangkok** | 2.324 | 2.308 | 0.342 | 0.196 | 2.543 | 2.544 | 2.525 | 2.509 | 2.512 | 2.500 | 2.492 | 2.488 | 2.438 | 2.449 | 2.689 | 2.683 | 2.614 | 2.587 | 2.604 | 2.580 |
| **Chachoengsao** | 0.852 | 0.852 | 0.176 | 0.122 | 0.799 | 0.793 | 0.785 | 0.797 | 0.771 | 0.776 | 0.754 | 0.763 | 0.699 | 0.742 | 0.649 | 0.698 | 0.613 | 0.705 | 0.622 | 0.682 |
| **Samut Prakan** | 0.651 | 0.652 | 0.156 | 0.092 | 0.776 | 0.763 | 0.759 | 0.753 | 0.738 | 0.739 | 0.724 | 0.727 | 0.679 | 0.701 | 0.818 | 0.826 | 0.785 | 0.802 | 0.813 | 0.795 |
| **Ratchaburi** | 0.798 | 0.790 | 0.166 | 0.091 | 0.715 | 0.704 | 0.709 | 0.715 | 0.703 | 0.700 | 0.698 | 0.693 | 0.673 | 0.684 | 0.656 | 0.691 | 0.622 | 0.687 | 0.647 | 0.682 |
| **Chon Buri** | 0.755 | 0.766 | 0.170 | 0.097 | 0.764 | 0.760 | 0.761 | 0.767 | 0.746 | 0.753 | 0.740 | 0.740 | 0.717 | 0.725 | 0.737 | 0.762 | 0.726 | 0.763 | 0.754 | 0.754 |
| **Rayong** | 2.120 | 2.093 | 0.301 | 0.155 | 2.310 | 2.302 | 2.291 | 2.267 | 2.274 | 2.262 | 2.254 | 2.254 | 2.201 | 2.212 | 2.427 | 2.413 | 2.346 | 2.318 | 2.331 | 2.312 |
| **Surat Thani** | 0.566 | 0.563 | 0.168 | 0.088 | 0.598 | 0.570 | 0.583 | 0.562 | 0.570 | 0.551 | 0.569 | 0.542 | 0.558 | 0.536 | 0.651 | 0.634 | 0.598 | 0.581 | 0.647 | 0.592 |
| **Phuket** | 0.978 | 0.971 | 0.201 | 0.113 | 1.000 | 1.014 | 1.007 | 1.004 | 0.996 | 1.015 | 1.003 | 1.024 | 1.011 | 1.028 | 1.162 | 1.153 | 1.112 | 1.076 | 1.148 | 1.106 |
| **Songkhla** | 1.241 | 1.222 | 0.192 | 0.102 | 1.044 | 1.000 | 1.054 | 1.030 | 1.054 | 1.022 | 1.067 | 1.020 | 1.069 | 1.014 | 1.000 | 0.981 | 1.026 | 1.036 | 1.093 | 1.026 |
| **Yala** | 1.311 | 1.313 | 0.213 | 0.109 | 1.125 | 1.089 | 1.129 | 1.111 | 1.133 | 1.099 | 1.149 | 1.092 | 1.170 | 1.089 | 1.019 | 1.030 | 1.043 | 1.062 | 1.062 | 1.044 |
| **Narathiwat** | 1.063 | 1.074 | 0.198 | 0.105 | 0.908 | 0.880 | 0.905 | 0.893 | 0.904 | 0.881 | 0.912 | 0.873 | 0.915 | 0.865 | 0.800 | 0.816 | 0.785 | 0.830 | 0.809 | 0.812 |

M1 = The model that use only previous incidence for feature selection.

M2 = The model that use previous incidence, meteorological, and particulate matter for feature selection.

(node)= The number of nodes of 1 layer. (node, node) = The number of nodes of 2 layer.

**Table S4** The average of MAE of test set for universal model.

| **Province** | **LR_M1** | **LR_M2** | **XGBoost_M1** | **XGBoost_M2** | **ANN_M1 (8)** | **ANN_M2 (8)** | **ANN_M1 (16)** | **ANN_M2 (16)** | **ANN_M1 (32)** | **ANN_M2 (32)** | **ANN_M1 (64)** | **ANN_M2 (64)** | **ANN_M1 (128)** | **ANN_M2 (128)** | **ANN_M1 (8,8)** | **ANN_M2 (8,8)** | **ANN_M1 (16,16)** | **ANN_M2 (16,16)** | **ANN_M1 (32,32)** | **ANN_M2 (32,32)** |
| --- | --- | --- | --- | --- | --- | --- | --- | --- | --- | --- | --- | --- | --- | --- | --- | --- | --- | --- | --- | --- |
| **Chiang Rai** | 1.428 | 1.420 | 1.820 | 2.065 | 1.824 | 1.837 | 1.829 | 1.816 | 1.787 | 1.824 | 1.784 | 1.824 | 1.716 | 1.847 | 1.960 | 1.925 | 1.915 | 1.886 | 2.011 | 1.927 |
| **Mae Hong Son** | 1.958 | 1.928 | 1.767 | 2.481 | 1.699 | 1.640 | 1.784 | 1.719 | 1.792 | 1.768 | 1.820 | 1.831 | 1.797 | 1.835 | 2.043 | 1.938 | 2.119 | 2.039 | 2.336 | 2.124 |
| **Chiang Mai** | 5.525 | 5.488 | 7.298 | 6.438 | 6.314 | 6.334 | 6.211 | 6.225 | 6.138 | 6.188 | 6.134 | 6.148 | 6.048 | 6.141 | 6.244 | 6.280 | 6.017 | 6.065 | 5.969 | 5.991 |
| **Nan** | 1.632 | 1.604 | 2.320 | 2.399 | 2.229 | 2.263 | 2.208 | 2.209 | 2.171 | 2.236 | 2.159 | 2.221 | 2.063 | 2.191 | 2.288 | 2.337 | 2.190 | 2.209 | 2.239 | 2.218 |
| **Lamphun** | 2.598 | 2.591 | 3.360 | 3.690 | 3.031 | 3.042 | 2.977 | 3.005 | 2.924 | 2.970 | 2.919 | 2.951 | 2.814 | 2.990 | 3.160 | 3.101 | 2.993 | 2.987 | 2.942 | 2.921 |
| **Lampang** | 2.865 | 2.856 | 2.718 | 2.814 | 3.233 | 3.246 | 3.193 | 3.188 | 3.164 | 3.178 | 3.145 | 3.174 | 3.090 | 3.123 | 3.251 | 3.287 | 3.096 | 3.134 | 3.075 | 3.126 |
| **Phrae** | 2.009 | 1.968 | 2.282 | 2.284 | 1.654 | 1.637 | 1.719 | 1.686 | 1.759 | 1.707 | 1.757 | 1.754 | 1.700 | 1.723 | 2.014 | 1.904 | 1.997 | 1.933 | 2.124 | 1.972 |
| **Khon Kaen** | 2.032 | 1.969 | 1.980 | 2.614 | 1.831 | 1.817 | 1.920 | 1.889 | 1.974 | 1.968 | 2.005 | 2.052 | 1.960 | 2.087 | 2.270 | 2.161 | 2.294 | 2.237 | 2.484 | 2.334 |
| **Nakhon Sawan** | 2.627 | 2.610 | 3.329 | 3.024 | 3.114 | 3.157 | 2.955 | 2.988 | 2.882 | 2.921 | 2.814 | 2.824 | 2.793 | 2.733 | 3.018 | 3.078 | 2.695 | 2.741 | 2.599 | 2.677 |
| **Nakhon Ratchasima** | 8.014 | 8.093 | 8.206 | 8.086 | 4.231 | 4.294 | 4.115 | 4.150 | 4.047 | 4.119 | 3.969 | 4.041 | 3.849 | 3.948 | 4.038 | 4.194 | 3.769 | 3.860 | 3.627 | 3.809 |
| **Phra Nakhon Si Ayutthaya** | 1.570 | 1.546 | 2.120 | 2.275 | 1.676 | 1.677 | 1.655 | 1.652 | 1.614 | 1.655 | 1.606 | 1.642 | 1.586 | 1.645 | 1.756 | 1.783 | 1.686 | 1.723 | 1.725 | 1.724 |
| **Bangkok** | 8.253 | 8.273 | 8.039 | 7.793 | 9.224 | 9.290 | 9.062 | 9.097 | 8.994 | 9.026 | 8.917 | 8.925 | 8.836 | 8.766 | 9.050 | 9.195 | 8.687 | 8.745 | 8.465 | 8.639 |
| **Chachoengsao** | 2.084 | 2.115 | 2.283 | 2.382 | 2.737 | 2.761 | 2.626 | 2.637 | 2.551 | 2.596 | 2.495 | 2.541 | 2.461 | 2.446 | 2.638 | 2.774 | 2.383 | 2.456 | 2.394 | 2.443 |
| **Samut Prakan** | 1.302 | 1.267 | 1.899 | 2.146 | 1.789 | 1.791 | 1.702 | 1.679 | 1.655 | 1.669 | 1.613 | 1.663 | 1.561 | 1.603 | 1.859 | 1.886 | 1.684 | 1.691 | 1.762 | 1.734 |
| **Ratchaburi** | 1.629 | 1.622 | 2.015 | 2.425 | 1.409 | 1.383 | 1.380 | 1.376 | 1.396 | 1.390 | 1.379 | 1.421 | 1.363 | 1.410 | 1.753 | 1.676 | 1.555 | 1.552 | 1.643 | 1.610 |
| **Chon Buri** | 1.249 | 1.212 | 1.828 | 1.607 | 1.393 | 1.399 | 1.317 | 1.326 | 1.267 | 1.308 | 1.256 | 1.292 | 1.248 | 1.260 | 1.451 | 1.476 | 1.254 | 1.316 | 1.371 | 1.339 |
| **Rayong** | 6.740 | 6.806 | 7.012 | 6.709 | 7.725 | 7.806 | 7.563 | 7.609 | 7.492 | 7.549 | 7.394 | 7.444 | 7.289 | 7.302 | 7.557 | 7.715 | 7.215 | 7.277 | 6.989 | 7.190 |
| **Surat Thani** | 1.292 | 1.262 | 1.960 | 1.771 | 1.458 | 1.448 | 1.415 | 1.406 | 1.396 | 1.440 | 1.420 | 1.479 | 1.367 | 1.505 | 1.754 | 1.697 | 1.613 | 1.613 | 1.760 | 1.654 |
| **Phuket** | 2.621 | 2.648 | 4.125 | 3.339 | 3.105 | 3.162 | 2.920 | 2.996 | 2.889 | 2.926 | 2.821 | 2.859 | 2.867 | 2.785 | 3.153 | 3.223 | 2.692 | 2.725 | 2.527 | 2.709 |
| **Songkhla** | 2.269 | 2.230 | 2.901 | 1.122 | 1.661 | 1.600 | 1.784 | 1.710 | 1.850 | 1.763 | 1.890 | 1.830 | 1.875 | 1.810 | 1.926 | 1.844 | 2.031 | 1.975 | 2.188 | 2.016 |
| **Yala** | 2.925 | 2.903 | 3.184 | 1.880 | 1.881 | 1.818 | 2.027 | 1.968 | 2.115 | 2.038 | 2.176 | 2.139 | 2.201 | 2.169 | 2.352 | 2.191 | 2.488 | 2.387 | 2.715 | 2.473 |
| **Narathiwat** | 2.545 | 2.486 | 2.464 | 1.669 | 1.563 | 1.466 | 1.703 | 1.621 | 1.772 | 1.705 | 1.838 | 1.812 | 1.903 | 1.837 | 2.057 | 1.908 | 2.121 | 2.073 | 2.419 | 2.177 |

M1 = The model that use only previous incidence for feature selection.

M2 = The model that use previous incidence, meteorological, and particulate matter for feature selection.

(node)= The number of nodes of 1 layer. (node, node) = The number of nodes of 2 layer.

**Figure S1.** Monthly influenza incidence (per 10,000 population in log scale) for 22 provinces from 2010 to 2019. The box and whisker are plotted. The gradient of color indicates the latitude from north to south of Thailand. Black dots indicate mean values, and yellow dots indicate outliers.

**Figure S2** Time series of influenza incidence for 22 provinces.


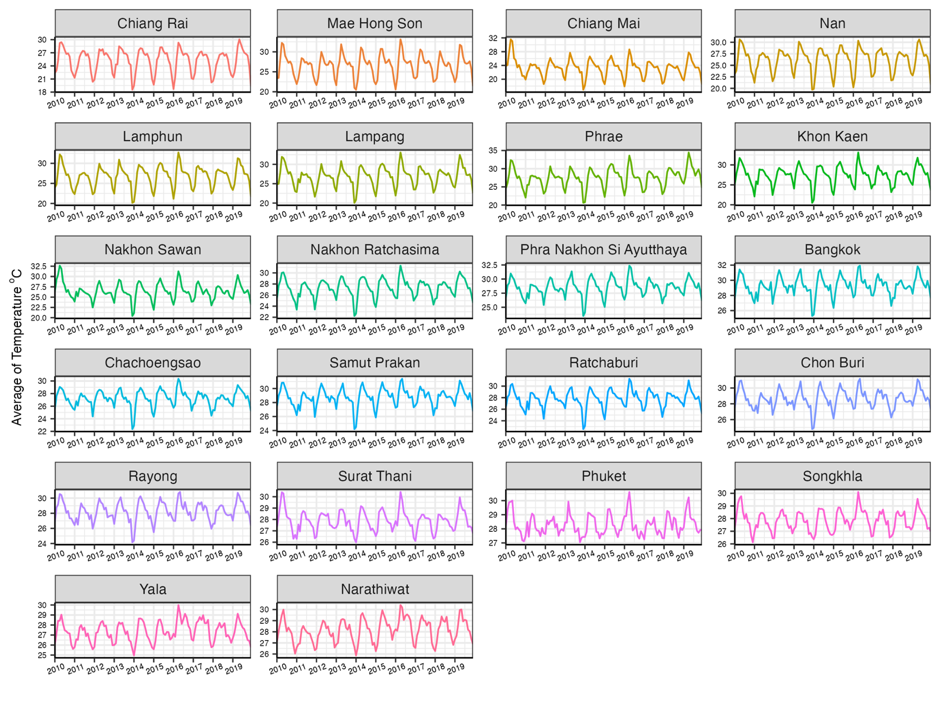


**Figure S3** Time series of average of temperature for 22 provinces.


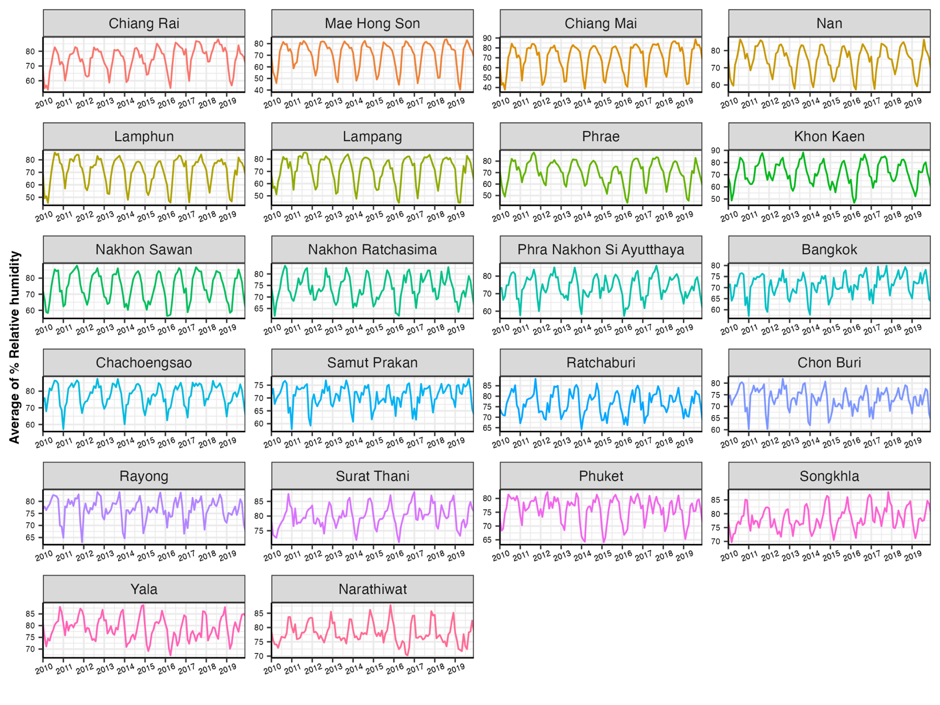


**Figure S4** Time series of relative humidity (%) for 22 provinces.


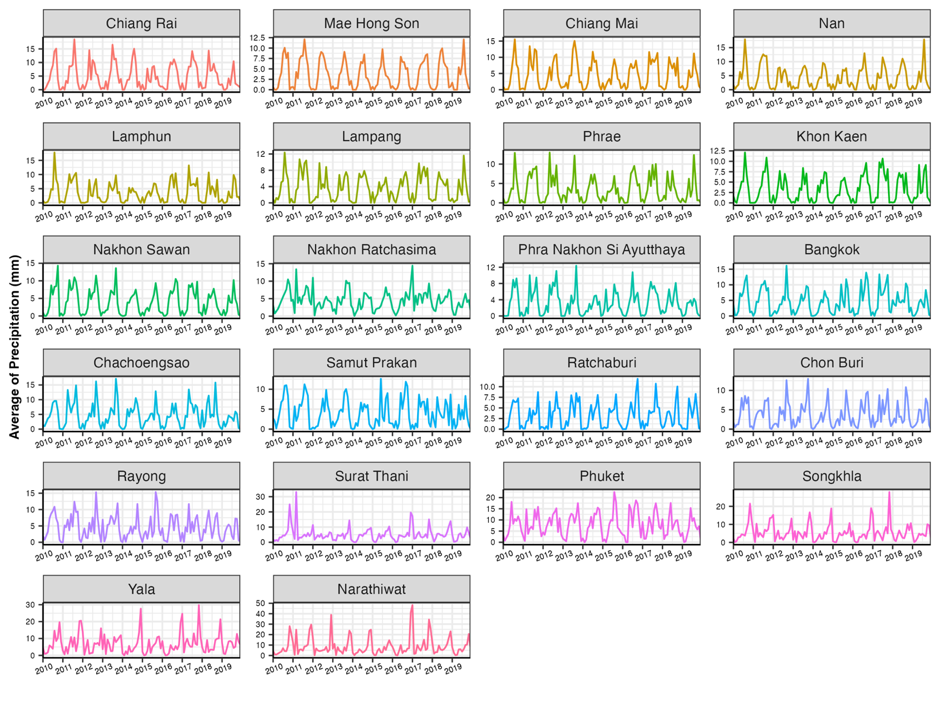


**Figure S5** Time series of precipitation for 22 provinces.

**
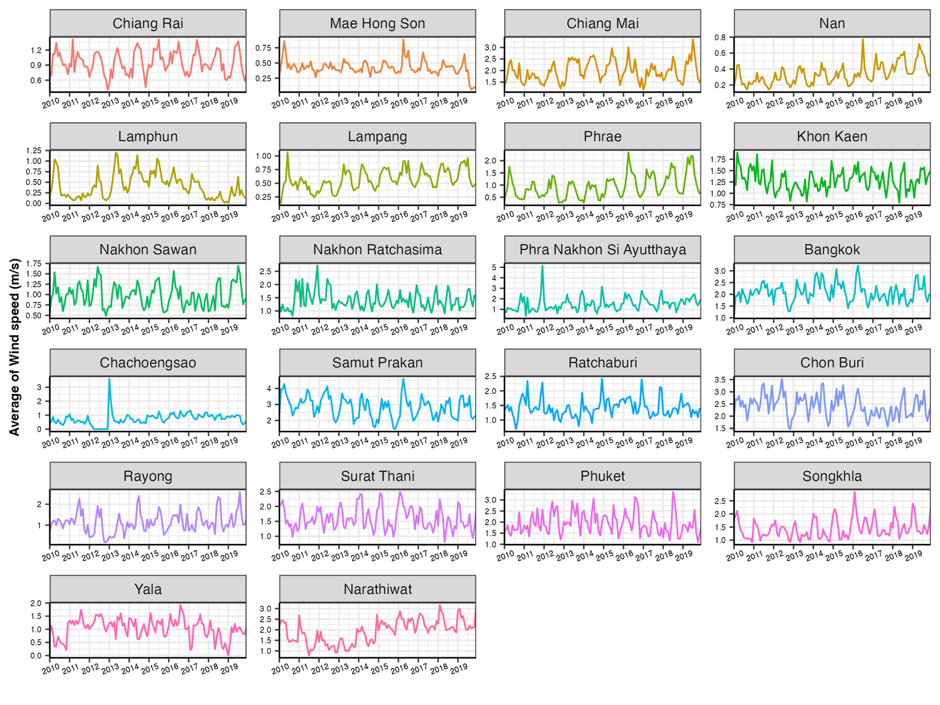
**

**Figure S6** Time series of wind speed for 22 provinces.

**
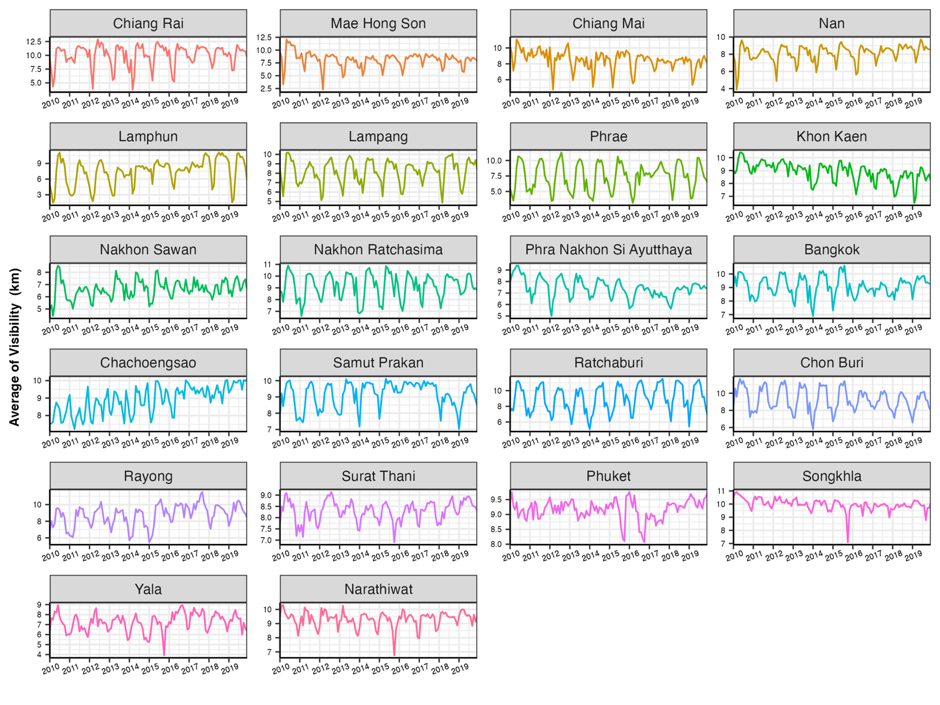
Figure S8** Time series of visibility for 22 provinces.

**
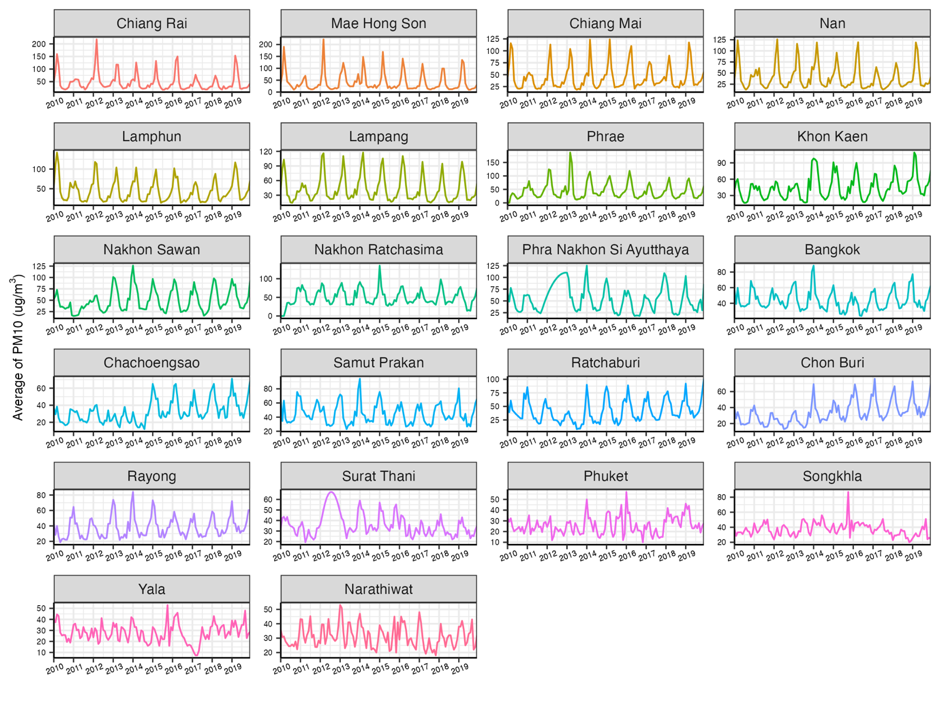
**

**Figure S8** Time series of PM10 for 22 provinces.

**
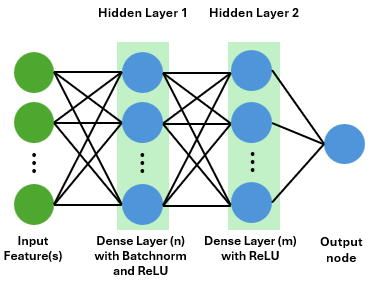
**

**Figure S9.** A typical architecture of a multi-layer ANN Model with two hidden layers. The input features are connected with two hidden layers where the number in the parenthesis denotes the number of nodes (hidden units) in that layer with ReLU activation functions. The first hidden layer includes Batch Normalization with $n$ nodes, followed by other layers with $m$ nodes, and finally, a single output node.


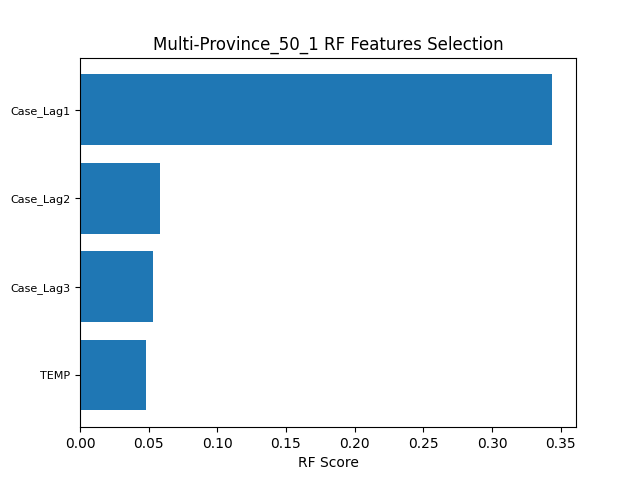

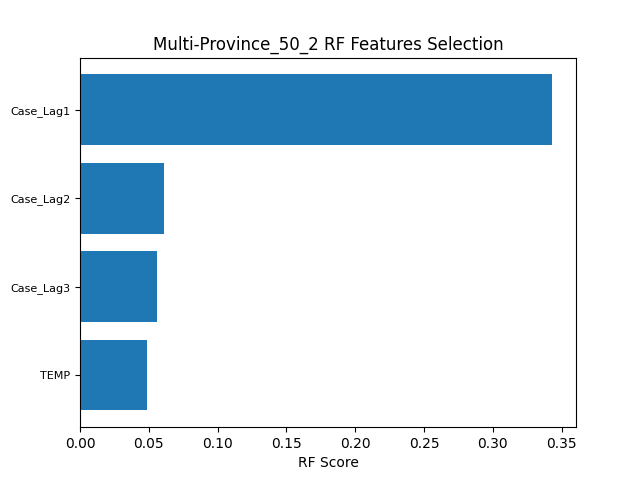

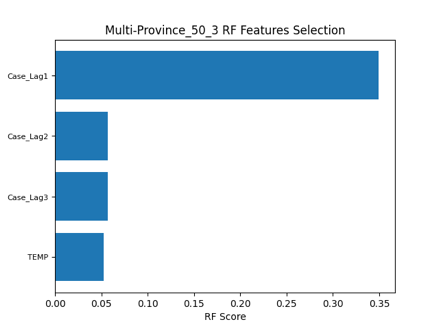

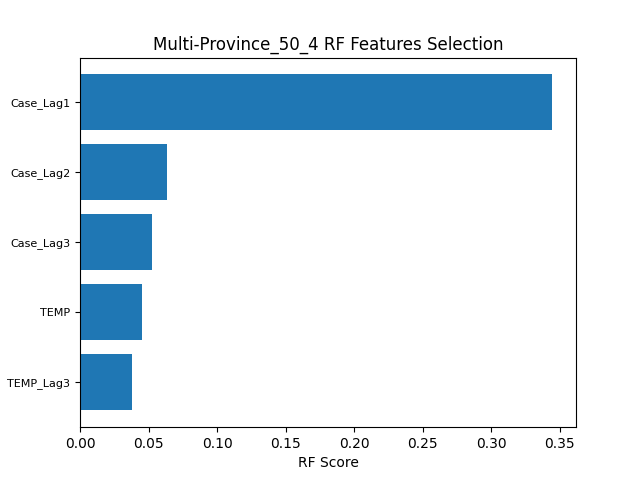

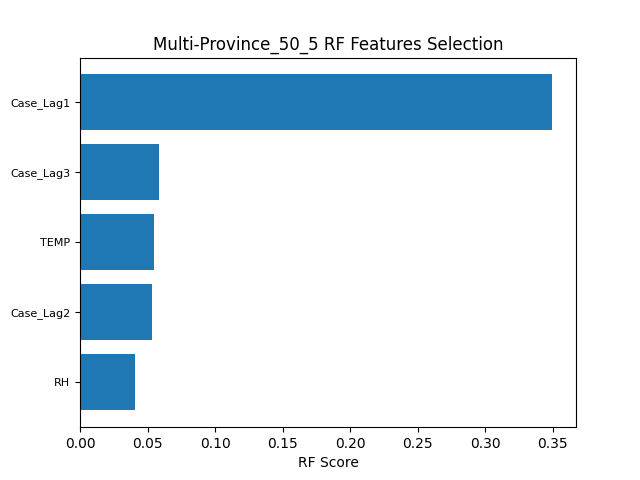

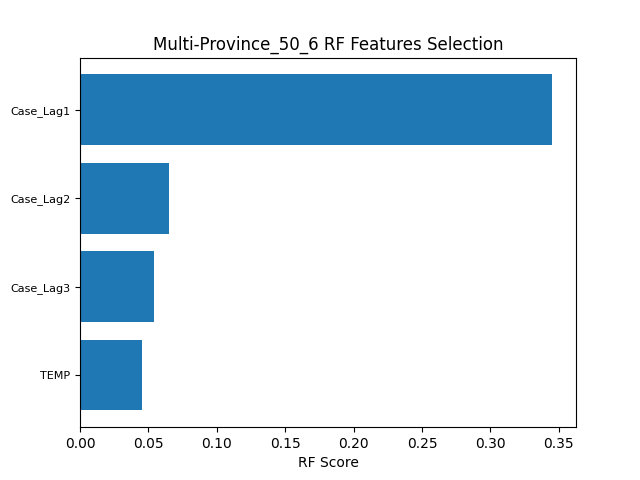


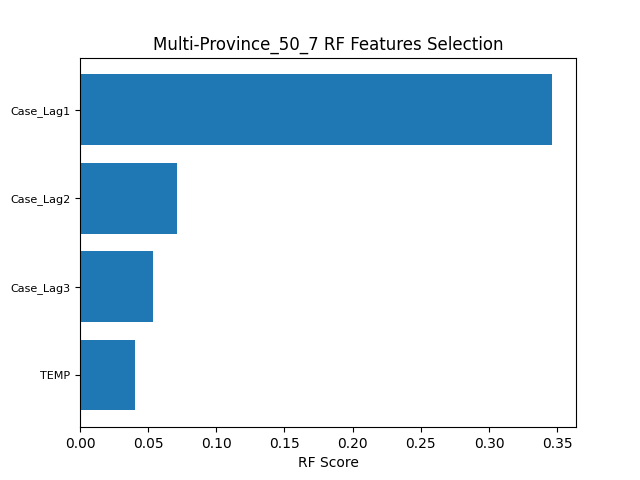

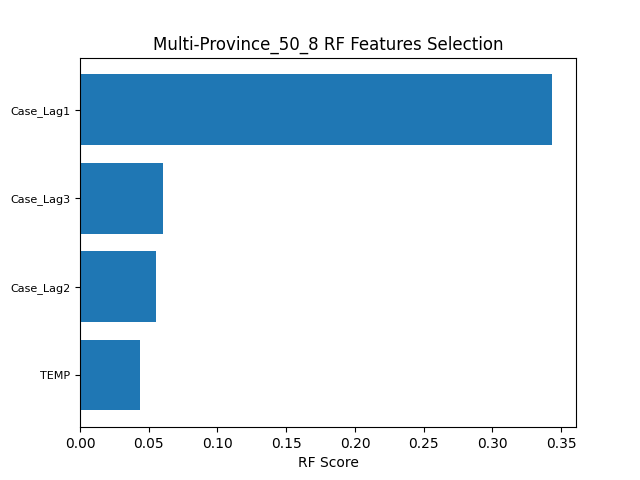

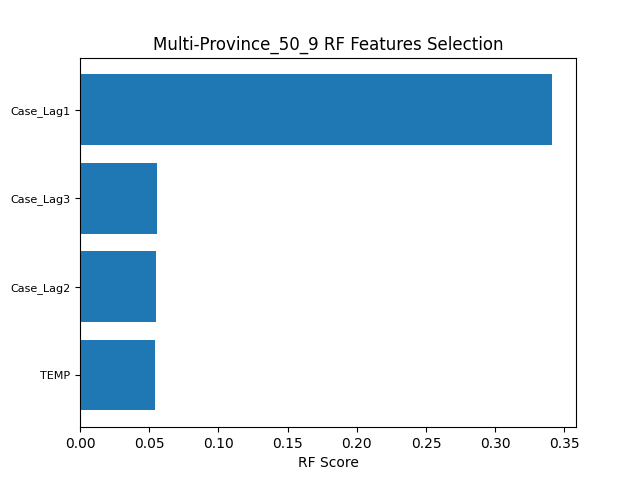

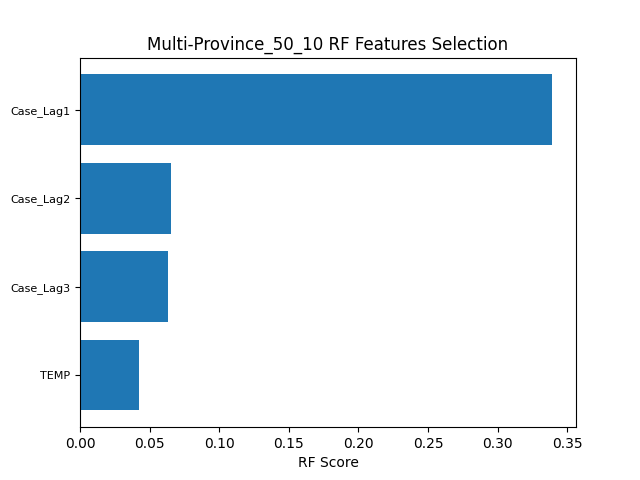

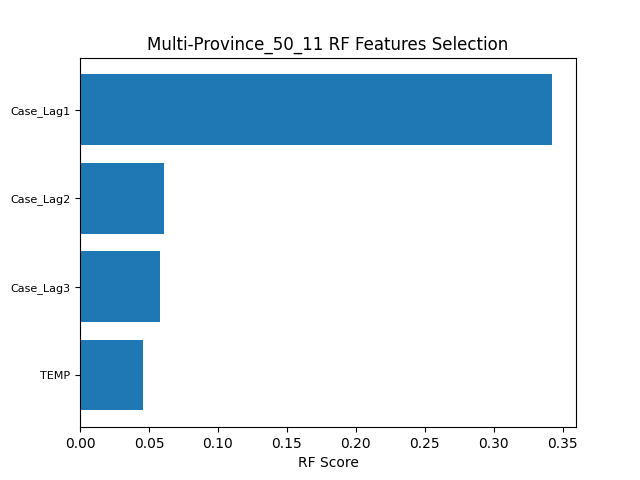

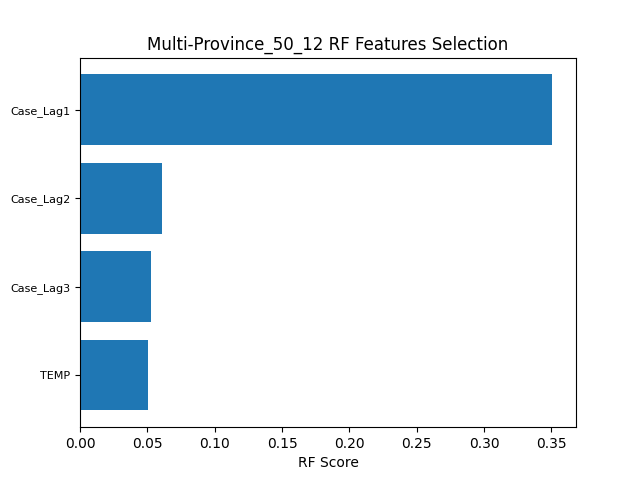


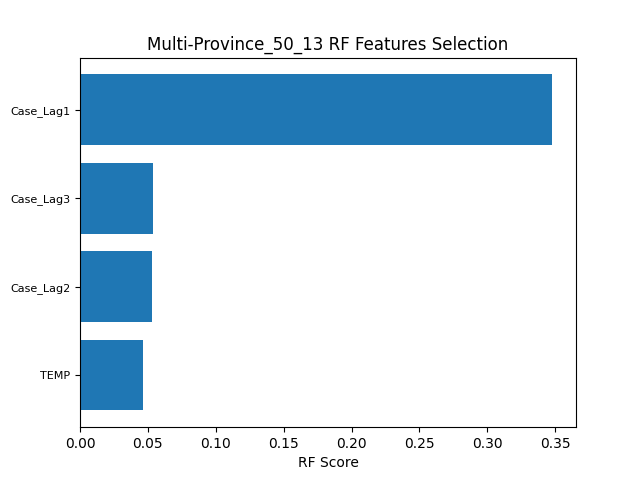

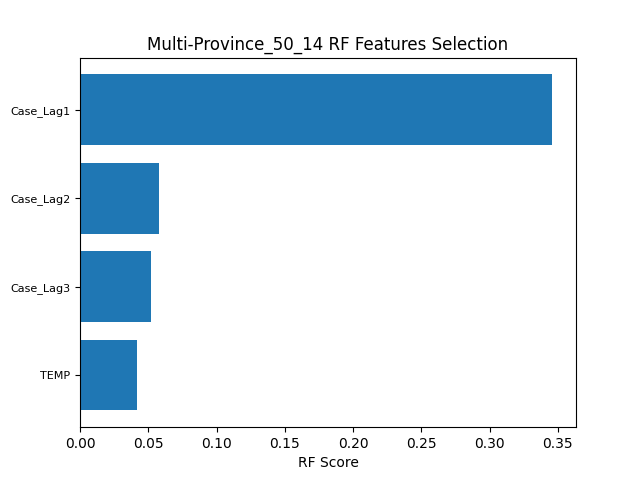

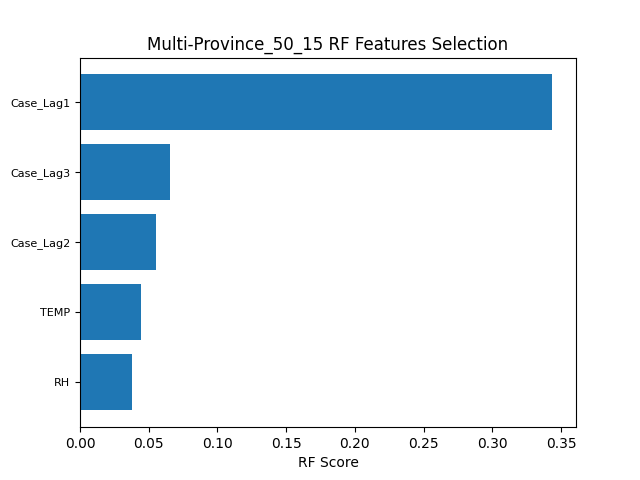


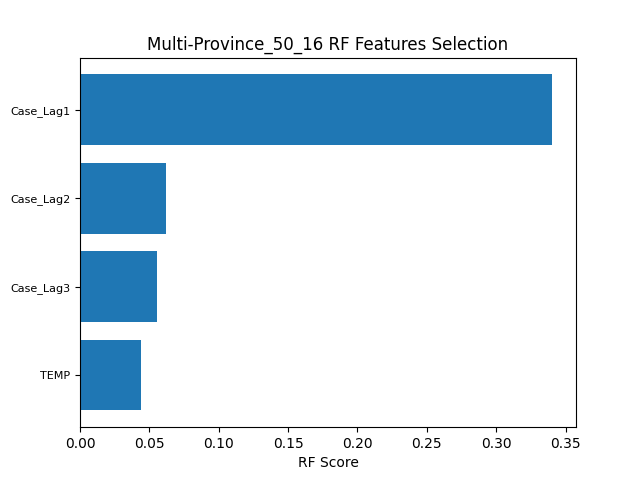

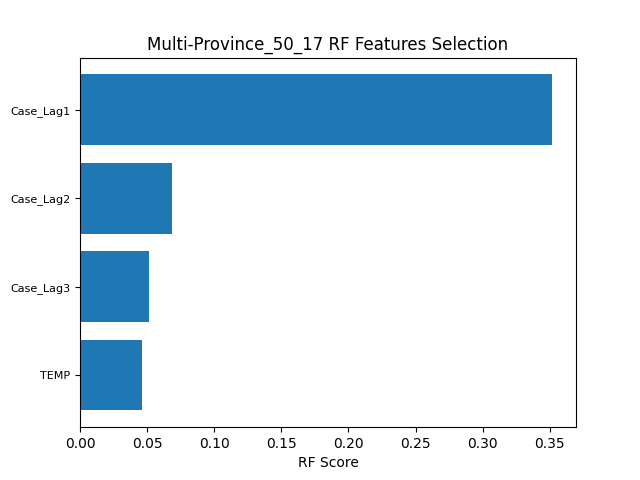

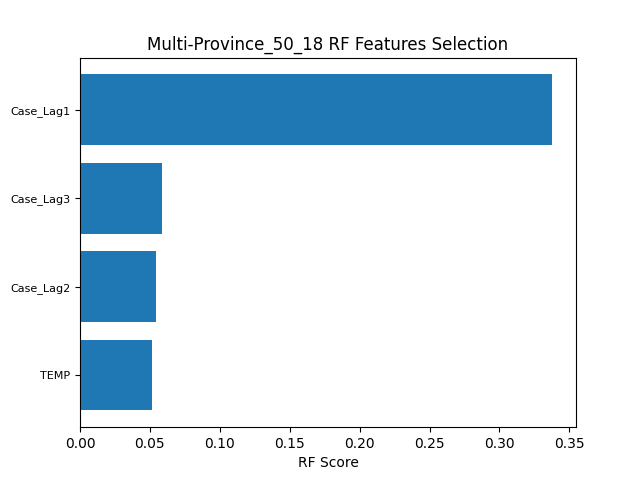


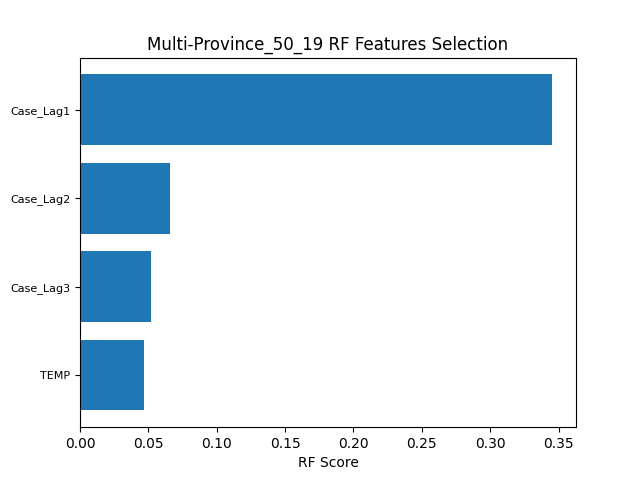

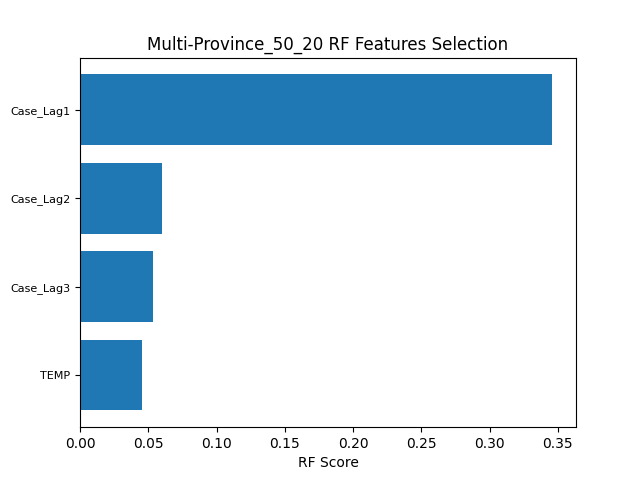

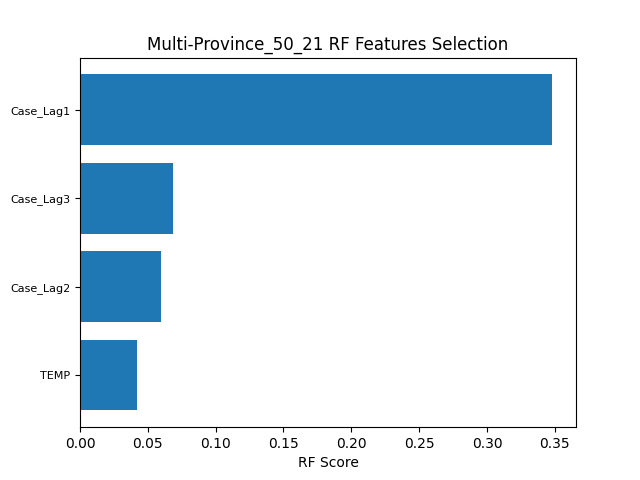

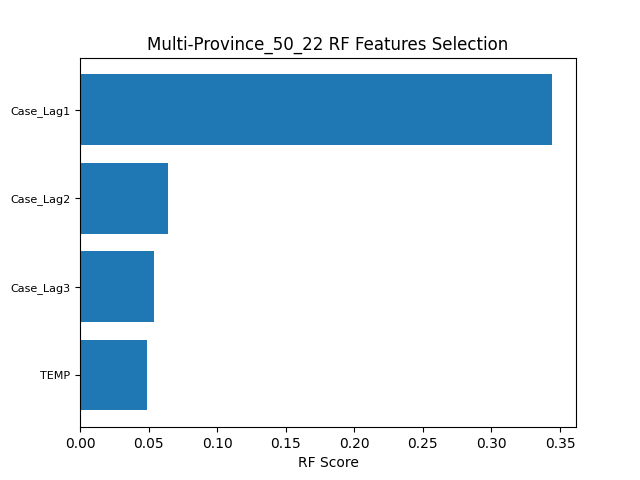

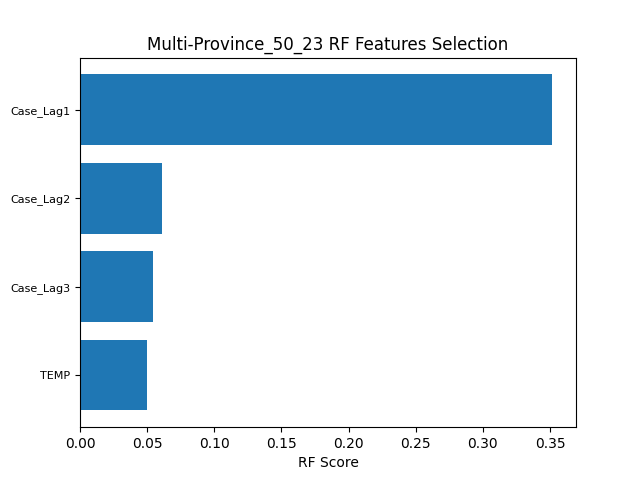

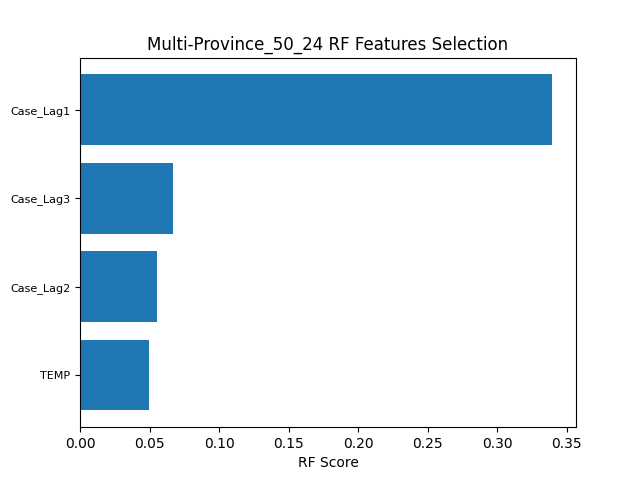

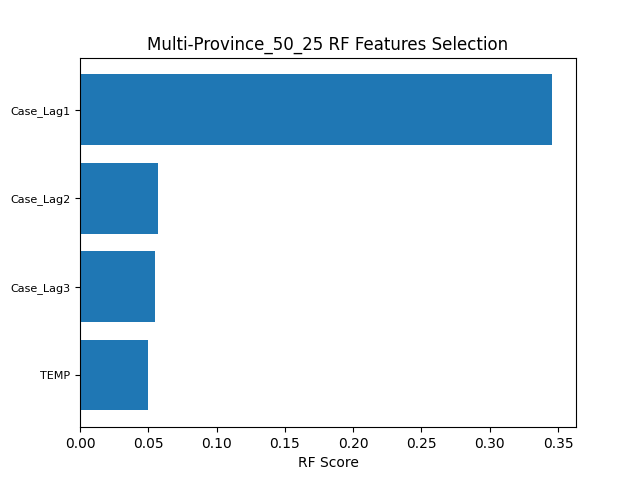

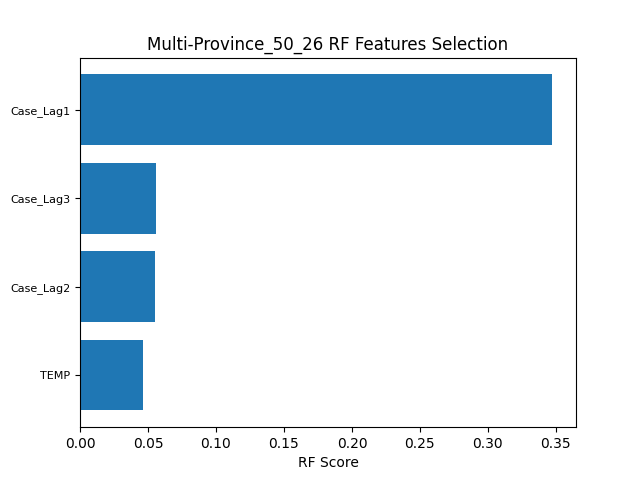

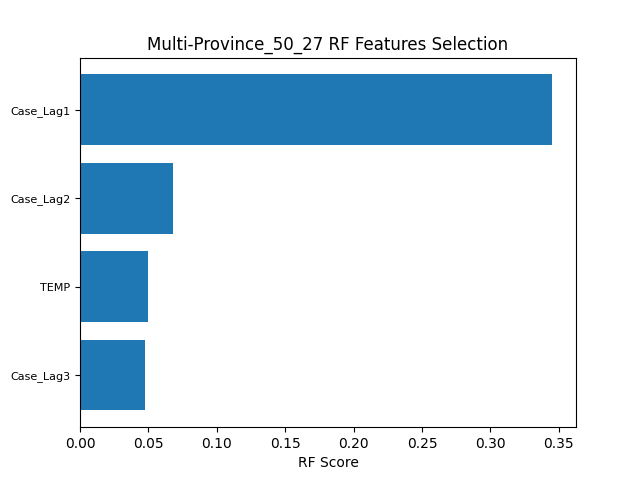

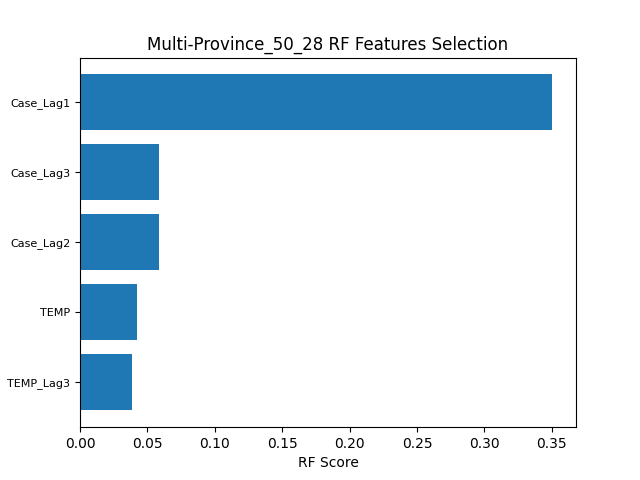

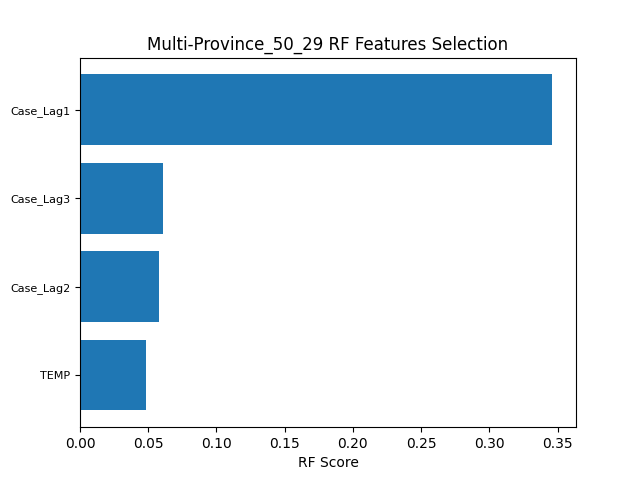

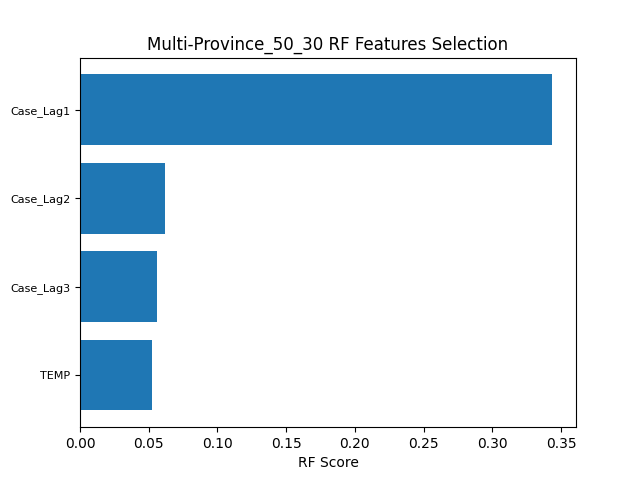

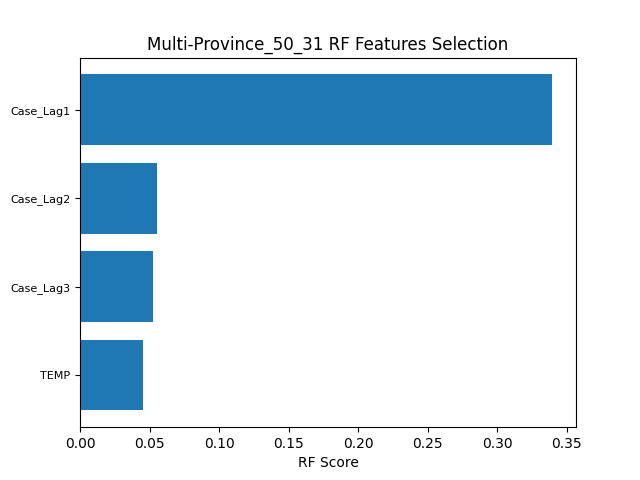

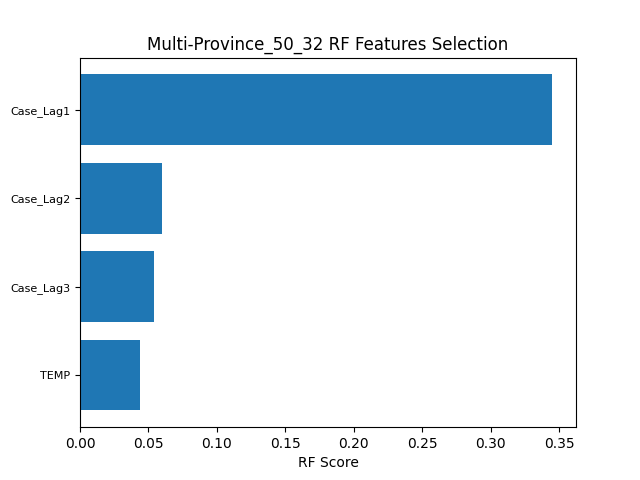

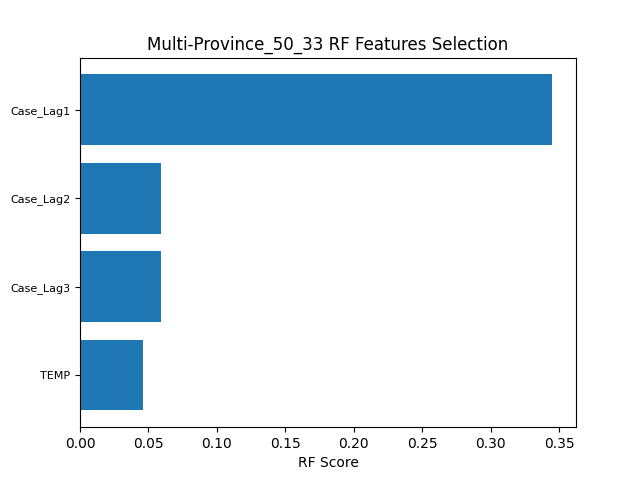

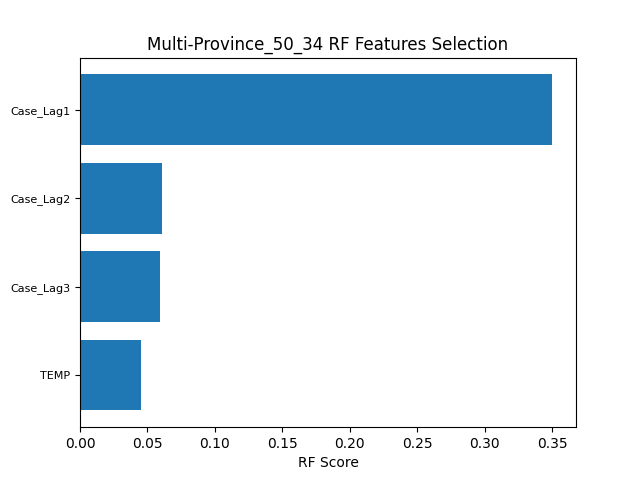

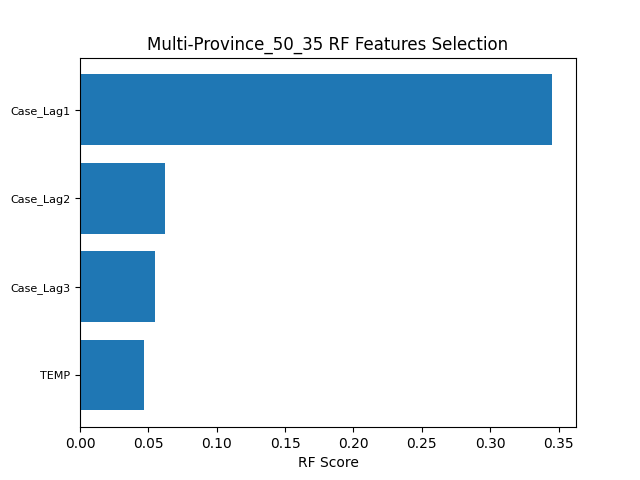

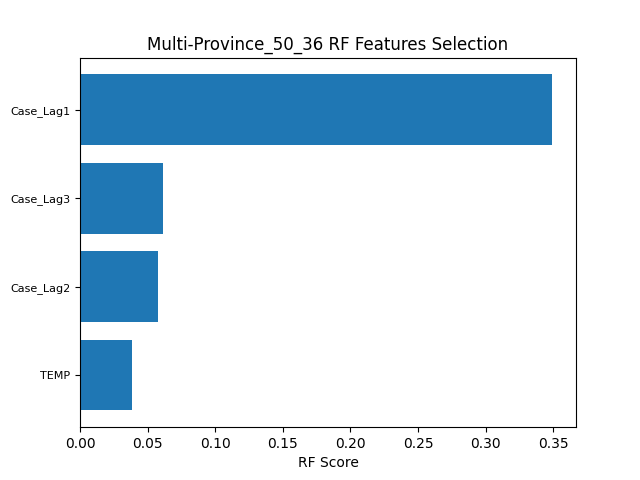

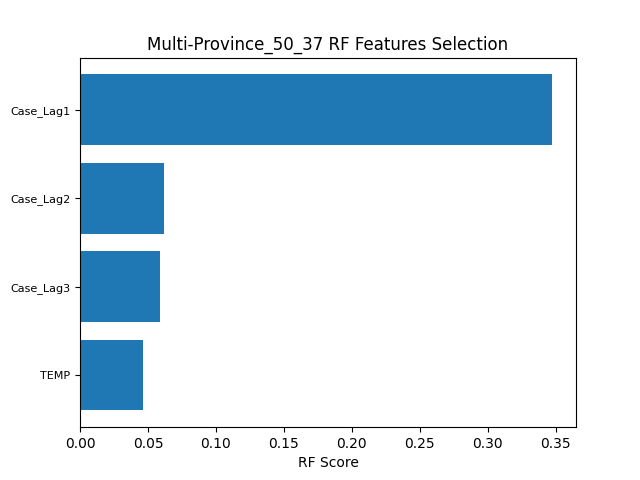

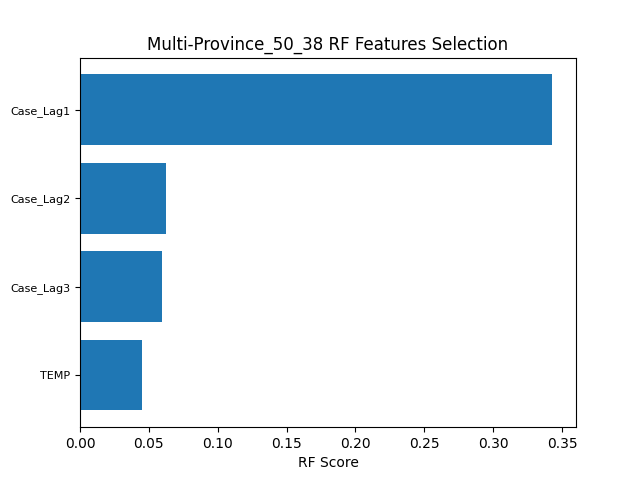

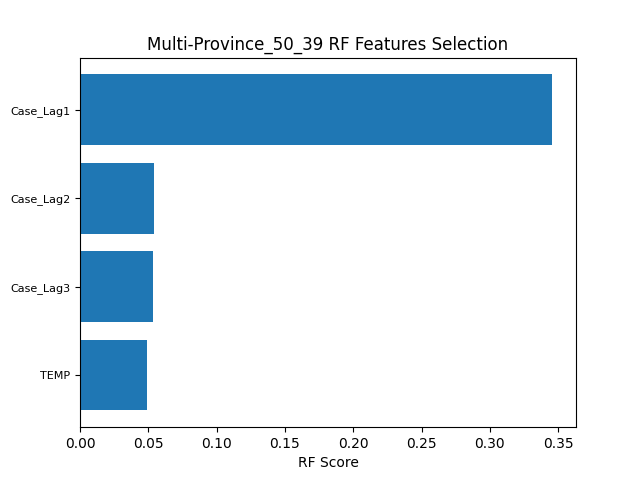

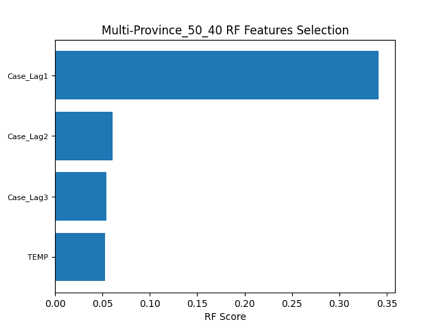

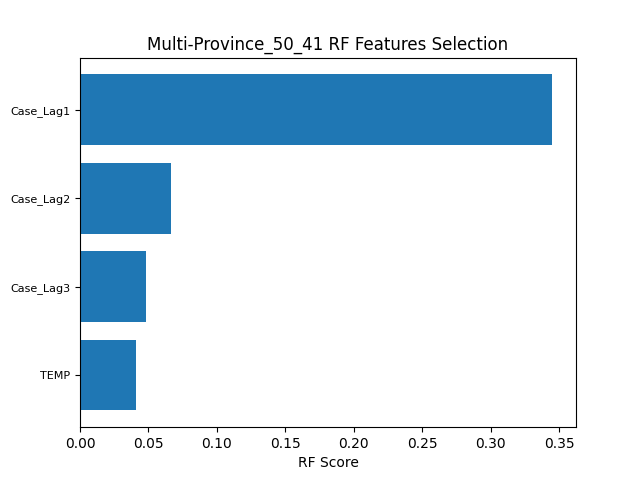

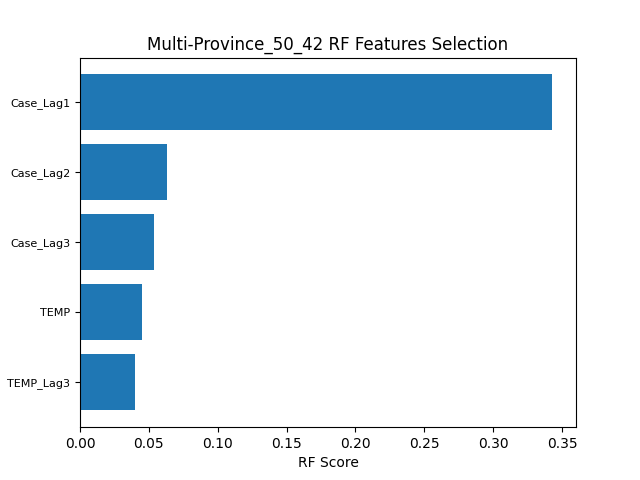

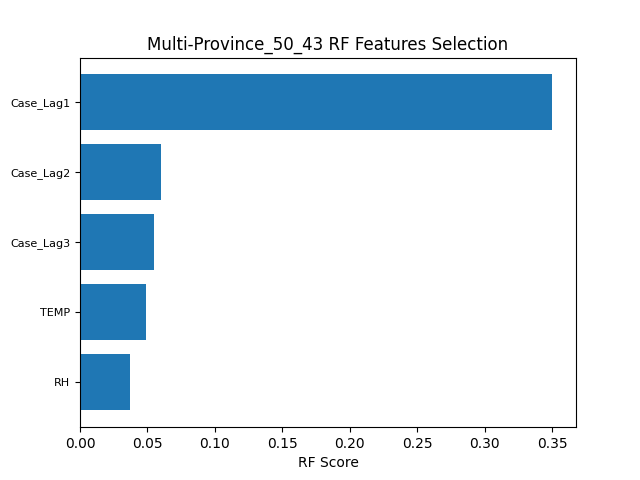

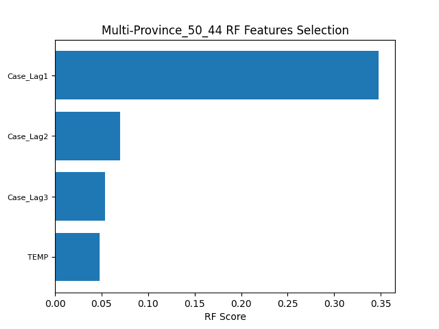

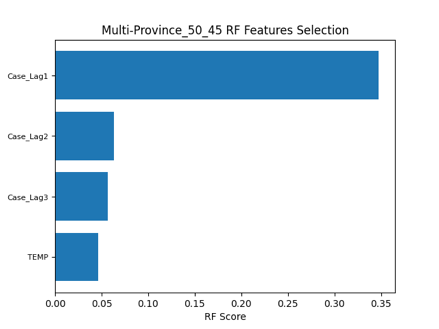

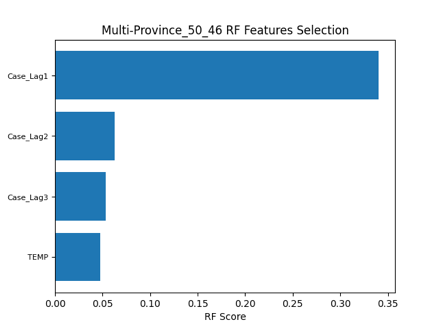

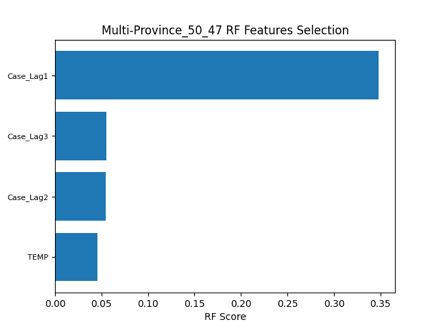

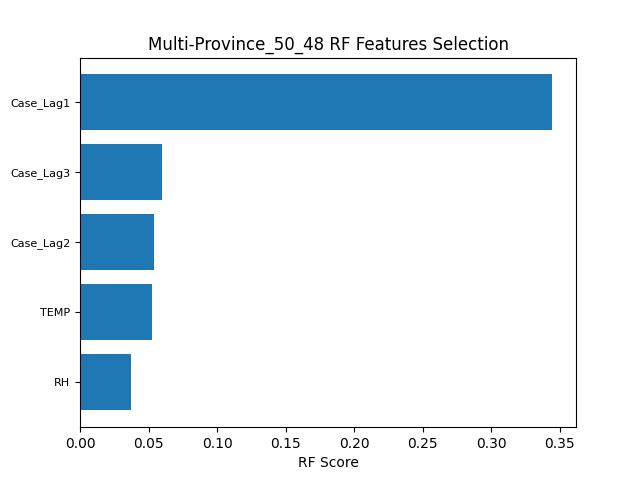

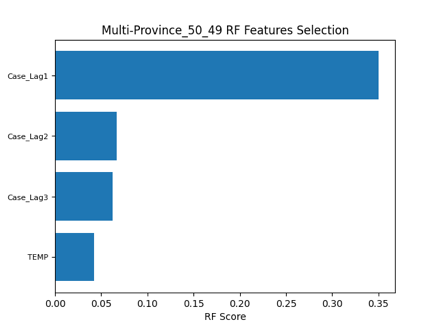

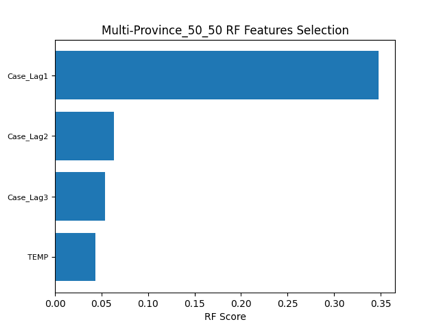


**Figure S10** Feature important rank of 22 provinces of universal model.

**Figure S11** Universal models with MAEs ranking. The ranking is based on the relative ranks from the lowest MAE across 50 runs for training (A) and testing (B) sets of the universal model for 22 provinces. The box-and-whisker plots are displayed, with black dots indicating mean values and black circles representing outliers. ($n$) represents the number of hidden units for 1 layer and ($n, n$) for 2 layers.

MAE = mean absolute error. M1 = The model that uses only the previous incidence for feature selection. M2 = The model that uses the previous incidence, meteorological, and particulate matter for feature selection.

**Figure S12.** The average of MAE of training (A) and test (B) sets for universal model over 50 runs. The bars represent the mean, and the error bars indicate the 95% confidence interval.

**Figure S13.** Average fitted and prediction incidence from 50 runs of models, where the grey areas indicate the prediction. The black lines indicate the actual incidence, while the red and blue lines indicate the predicted incidence from the M1 (16,16) and M2 (16,16) models, respectively. The colored highlights represent the 95% confidence intervals.

**Figure S14** The rank of MAEs for training (A) and test (B) sets for the transfer learning model for 50 runs. The box and whisker are plotted. Black dots indicate mean values and black circles indicate outliers.

**Figure S15** The average of MAE of the training (A) and test (B) sets for the transfer learning model for 50 experiment runs. The bars indicate the mean, and the error bars indicate the 95% confidence interval.
